# Purification-free antifungal biocontrol platform using engineered *Saccharomyces cerevisiae* secreting *Trichoderma atroviride* chitinase

**DOI:** 10.1186/s40643-026-01054-z

**Published:** 2026-04-29

**Authors:** Ha-Yeon Song, Dae-Hyuk Kim, Jung-Mi Kim, Ji Young Kang

**Affiliations:** 1 Institute of Life Science and Natural Resources, Wonkwang University, Iksan, 54538 Republic of Korea; 2 Department of Bioactive Material Sciences, Institute for Molecular Biology and Genetics, Jeonbuk National University, Jeonju, 54896 Republic of Korea; 3 Department of Biomedical Materials Science, Jeonbuk Advanced Bio-Convergence Academy, Wonkwang University, Iksan, 54538 Republic of Korea; 4 Industrial Microbiology and Bioprocess Research Center, Korea Research Institute of Bioscience and Biotechnology (KRIBB), Jeongeup, 56212 Republic of Korea

**Keywords:** *Saccharomyces cerevisiae*, *Trichoderma atroviride*, Chitinase, Yeast secretion, Antifungal biocontrol, Extracellular enzyme production

## Abstract

Chitinases from *Trichoderma* species exhibit strong antifungal activity and high biocontrol potential, yet their industrial utilization has been constrained by low heterologous secretion efficiency and costly purification processes. In this study, the Generally Recognized As Safe yeast *Saccharomyces cerevisiae* Y2805 was engineered to secrete chitinase Tch36 from a Korean isolate of *T. atroviride*, using a rice α-amylase signal peptide under a constitutive glyceraldehyde-3-phosphate dehydrogenase (GPD) promoter. When cultivated in glycerol–colloidal chitin medium, the recombinant yeast exhibited approximately a fivefold increase in measurable chitinase activity relative to the SC mock control, whereas the empty-vector control showed only a modest increase. The crude, non-concentrated culture filtrate (approximately 1000 U L⁻^1^) displayed statistically significant antifungal activity against 12 fungal species, including 9 phytopathogens and 3 opportunistic *Aspergillus* species. Among the phytopathogens, *Fusarium graminearum* was one of the most strongly inhibited species, showing approximately 40–50% suppression of colonial growth on solid medium. In liquid culture, microscopy-based germination assays revealed ≥ 63% inhibition of early hyphal elongation in *Botrytis cinerea* and *A. niger*. All antifungal effects were evaluated relative to filtrates from the empty-vector control prepared under equivalent dilution conditions. The culture filtrate also enhanced protoplast formation, providing direct evidence of chitinase-associated cell wall weakening. Collectively, these results establish a purification-free, yeast-based heterologous secretion platform capable of producing active *Trichoderma* chitinase with inhibitory effects on diverse plant- and animal-associated fungi. This strategy has practical potential for biocontrol applications and for sustainable bioprocessing technologies based on microbial chitinase production.

**Graphical abstract:**

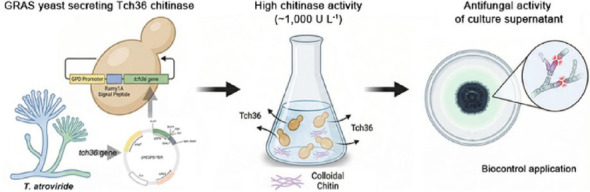

**Supplementary Information:**

The online version contains supplementary material available at 10.1186/s40643-026-01054-z.

## Introduction

Fungal pathogens that infect plants and animals, including humans, cause substantial economic losses and pose a major threat to food security and ecosystem stability (Fisher et al. 2020). Chemical fungicides have long been the dominant control strategy; however, their repeated use has led to the emergence of resistant fungal strains and severe contamination of soil and water (Swiontek Brzezinska et al. 2014; El-Sayed et al. 2024). Consequently, there is an urgent need for sustainable and biosafe antifungal solutions that can be practically implemented under controlled production conditions.

Among biological alternatives, chitinases (enzymes that hydrolyze the β-1,4-glycosidic bonds in chitin, a key structural polymer of the cell walls of fungi) have gained considerable attention for their strong antifungal activity and biocontrol potential (Deng et al. 2019; Li et al. 2018). Beyond pathogen control, chitinases also serve important roles in ecofriendly bioprocessing, such as converting crustacean shell waste into valuable oligosaccharides and chitin derivatives (Wang et al. 2020a, b). Within this enzyme family, *Trichoderma* species are particularly notable for producing multiple chitinases with high catalytic efficiency and broad substrate specificity, making them widely recognized as effective biological control agents (Benítez et al. 2004). These fungi are among the most studied biocontrol organisms due to their ability to antagonize a wide range of plant pathogens through mechanisms such as mycoparasitism, enzyme secretion, and induced systemic resistance (Harman et al. 2004; Druzhinina et al. 2011).

Nevertheless, the direct industrial use of filamentous fungi such as *Trichoderma* is often challenged by relatively slower growth rates, morphological complexity, and difficulties in maintaining stable enzyme secretion under large-scale fermentation conditions (Benítez et al. 2004; Kubicek et al. 2019). In large bioreactors, hyphal aggregation and variable oxygen transfer further complicate process control and scale-up, leading to inconsistent enzyme yields. These process-related limitations frequently result in high production and downstream processing costs, thereby reducing the economic feasibility of Trichoderma-based enzyme production. Moreover, *Trichoderma* species are not classified as Generally Recognized As Safe (GRAS) organisms, which restricts their application in food, feed, and certain agricultural formulations that require regulatory biosafety compliance (Gasser & Mattanovich 2018).

To overcome these limitations, heterologous expression systems have been developed in *Escherichia coli*, *Komagataella phaffii* (syn. *Pichia pastoris*), and *Saccharomyces cerevisiae* (Draborg et al. 1996; Pérez-Martínez et al. 2007; Adrangi and Faramarzi 2013). While *E. coli* remains the most frequently used host due to its rapid growth, it often fails to produce properly folded eukaryotic proteins, leading to insoluble inclusion bodies that require laborious refolding (Boer et al. 2007; Rosano and Ceccarelli 2014). The *K. phaffii* system has demonstrated strong secretory capability for fungal chitinases (Pérez-Martínez et al. 2007, 2014; Ahmad et al. 2014); however, it requires methanol-inducible promoters and is not GRAS-certified, raising safety concerns for open-environment applications (Cereghino and Cregg 2000; Ahmad et al. 2014). Additionally, extensive glycosylation in *K. phaffii* can alter protein structure and reduce the activity of hydrolytic enzymes such as chitinases (Ahmad et al. 2014).

Previous attempts to express *Trichoderma* chitinases in *Saccharomyces cerevisiae* have mainly focused on intracellular extracts rather than secreted forms (Draborg et al. 1996) or, like *Komagataella phaffii*–based systems, have required costly downstream purification (Adrangi and Faramarzi 2013). These studies highlight the persistent bottlenecks in secretion efficiency, extracellular recovery, and biosafety compliance. The economic impact of downstream processing is considerable, as purification steps account for up to 50–80% of total enzyme production costs (Shukla et al. 2010). This cost structure makes purified chitinase preparations prohibitively expensive for large-scale agricultural use, where low-cost, formulation-ready bioagents are essential. Consequently, the development of a system that allows extracellular production of functionally active chitinase in a GRAS host without the need for purification would represent a major step toward industrial feasibility.

The GRAS yeast *S. cerevisiae* offers a promising alternative due to its proven biosafety, eukaryotic secretion machinery, and established large-scale fermentation infrastructure (Stubbs et al. 2001; Gasser & Mattanovich 2018). Unlike *K. phaffii*, *S. cerevisiae* does not require toxic inducers and enables the secretion of functionally active proteins directly into the culture medium, thereby simplifying downstream processing (da Silva et al. 2016). Our research group has developed a secretion-optimized yeast platform using the episomal pYEGPD-TER vector system in *S. cerevisiae* Y2805, validating its robustness for efficient, signal peptide-guided secretion of diverse heterologous proteins (Kim et al. 2008, 2010; Shin et al. 2013; Nguyen et al. 2015; So et al. 2025).

In this study, we applied this optimized system to express the *tch36* gene from *Trichoderma atroviride*, which encodes the chitinase Tch36. Unlike previous studies focused on purified enzymes, we directly evaluated the antifungal efficacy of the crude, non-concentrated culture supernatant. By integrating a signal peptide-based secretion system with an inducer-free, environmentally benign yeast host, we established a purification-free extracellular production platform that enables direct functional evaluation of secreted chitinase under laboratory-scale conditions. This approach not only overcomes the biosafety and process limitations associated with filamentous fungal and *Pichia*-based systems but also presents a practical model for sustainable antifungal biocontrol and ecofriendly bioprocessing applications.

## Materials and methods

### Strains and culture conditions

The *chit36* gene was isolated from the filamentous fungus *T. atroviride* (KACC 40774) for heterologous expression. *E. coli* DH5α was used as the host for plasmid propagation, and *Saccharomyces cerevisiae* Y2805 (*MATα pep4::HIS3 prb1-δCan1 GAL2 his3 ura3-52*) was used as the host for yeast transformation (Kim et al. 2010).

For antifungal activity assays, a panel of nine plant pathogenic fungi and three animal pathogenic fungi was utilized. The plant pathogens included *Alternaria alternata* (KACC 45440), *Botrytis cinerea* (KACC 47009), *Botryosphaeria dothidea* (KACC 45481), *Colletotrichum acutatum* (KACC 40042), *Fusarium fujikuroi* (KACC 44002), *F. graminearum* (KACC 41044), *Rhizoctonia solani* AG-1(IA) (KACC 40101), *T. atroviride* (KACC 40777), and *T. viride* (KACC 43826). The animal pathogens were *Aspergillus fumigatus* (KCTC 6145), *A. flavus* (KCTC 6984), and *A. niger* (KCTC 6960). All fungal strains were obtained from the Korean Agricultural Culture Collection (KACC) and the Korean Collection for Type Cultures (KCTC), with their corresponding accession numbers indicated in parentheses.

The model filamentous fungus *A. nidulans* FGSC A4, obtained from the Fungal Genetics Stock Center (FGSC, Manhattan, USA), was used for both antifungal activity and protoplast formation assays. For the protoplast formation assay, *Aspergillus nidulans* FGSC4 (wild-type reference strain) and the pigment-deficient mutant strain WX (*npgA1*), genetically characterized by Han et al. (2005) and previously used in our study (Kim et al. 2015), were used.

### Plasmid construction

*T. atroviride* KACC 40774 was cultured in potato dextrose broth (PDB; BD Difco, Franklin Lakes, USA) at 30 °C for 3 days. Mycelia were harvested by filtration, and total RNA was extracted using TRIzol reagent (Thermo Fisher Scientific, Waltham, USA) according to the manufacturer’s instructions. First-strand cDNA synthesis was performed using SuperScript™ II Reverse Transcriptase (Invitrogen, Carlsbad, USA). The *tch36* cDNA was amplified via the polymerase chain reaction (PCR) using the gene-specific primers Ta-chit36F and Ta-chit36R, designed based on the *T. atroviride* reference sequence (GenBank accession no. KT992143). The PCR amplicon was purified cloned into the pGEM-T Easy vector (Promega, Madison, USA), then transformed into *Escherichia coli* DH5α competent cells. Recombinant clones were screened by restriction digestion and verified by Sanger sequencing (Solgent, Seoul, Korea).

For heterologous expression in *Saccharomyces cerevisiae*, the episomal shuttle vector pYEGPD-TER was used. This vector contains the constitutive glyceraldehyde-3-phosphate dehydrogenase (GPD) promoter and GAL7 terminator and is suitable for efficient recombinant protein secretion (Kim et al. 2010). To facilitate extracellular secretion of Tch36, the 31-amino-acid signal peptide from rice α-amylase 1A (Ramy1A; GenBank accession no. X16509) was fused to the 5′ end of the *tch36* coding region via fusion PCR (Ge et al. 1997; Kim et al. 2010). In the first round of PCR, the Ramy1A signal peptide was amplified using primers asp-F and asp-tchF-R or asp-tchM-R, while the *tch36* coding region was amplified either as a full-length form using primers Ftch36F-F and Ftch36F-R or as a mature form lacking the native N-terminal signal peptide using primers Ftch36M-F and Ftch36F-R. Two constructs were generated: a full-length version containing the native signal peptide and a mature version lacking the N-terminal 25-amino-acid signal sequence. The primer sets used for each construct are provided in Supplementary Table 1.

Fusion PCR products were gel-purified and re-amplified using the outer primers asp-F and Ftch36F-R, which introduced *BamHI* and *SalI* restriction sites at the 5′ and 3′ ends, respectively. Verified amplicons were cloned into pGEM-T Easy, sequence-confirmed, digested with *BamHI* and *SalI*, and subcloned into the corresponding sites of the episomal expression vector pYEGPD-TER. The resulting recombinant plasmids were propagated in *Escherichia coli* DH5α and purified using a plasmid miniprep kit (Promega). Purified plasmids were subsequently transformed into *Saccharomyces cerevisiae* Y2805 using the lithium acetate–PEG method, as described in the yeast transformation and screening section.

### Yeast transformation and screening

Recombinant pYEGPD-TER vectors harboring either the full-length (pYETch36F) or mature signal peptide-truncated (pYETch36M) forms of the *tch36* gene were introduced into *S. cerevisiae* Y2805 using the lithium acetate (LiAc) transformation method (Ito et al. 1983). Briefly, competent yeast cells were prepared and mixed with plasmid DNA and single-stranded carrier DNA in a transformation buffer containing LiAc and polyethylene glycol (PEG). The mixtures were heat-shocked at 42 °C for 15 min, spread onto synthetic complete (SC) agar plates lacking uracil (ura⁻), and incubated at 30 °C for 3 days. Transformants were selected based on auxotrophic complementation, and more than 100 colonies were obtained for each construct.

To confirm the presence of the *tch36* insert, colony PCR was performed on randomly selected transformants. A single colony was suspended in 20 μL sterile distilled water, and 1.5 μL suspension was used as the PCR template. The *tch36*-specific primers produced the expected 1.1-kb amplicon in both pYETch36F and pYETch36M transformants. Plasmid DNA used during transformation served as a positive control (PC), while wild-type Y2805 and an empty-vector transformant were used as negative controls (NCs). Amplicons were analyzed via electrophoresis on 1.2% agarose gels and visualized with ethidium bromide staining.

Then quantitative reverse transcription PCR (qRT-PCR) was performed to determine the transcript levels of the *tch36* gene in yeast transformants. For each strain, cultures were independently prepared on three separate occasions (*n* = 3), and total RNA was independently extracted from each culture. Total RNA was extracted using a Hybrid-R™ total RNA isolation kit (GeneAll, Seoul, Korea), treated with DNase I to eliminate genomic DNA contamination, and reverse-transcribed into cDNA using a reverse transcription system (Promega). qRT-PCR was conducted on a LightCycler® 96 System (Roche, Switzerland) using FastStart Essential DNA Green Master Mix (Roche) and gene-specific primers (Supplementary Table 1). Each qRT-PCR reaction was performed in technical triplicate. Transcript levels were normalized to the *S. cerevisiae* GPD housekeeping gene, and relative expression was calculated using the 2⁻ΔΔCt method, as described previously (So et al. 2025). The mean value obtained from each biological replicate was used as the statistical unit for analysis.

To verify protein-level expression of Tch36 further, total soluble protein extracts were prepared from selected transformants. Yeast cells were harvested, frozen in liquid nitrogen, and ground into a fine powder. Approximately 1.5 mL powdered biomass was transferred to a microcentrifuge tube, mixed with 600 µL extraction buffer (40 mM Tris–HCl, pH 7.5, supplemented with 1 × protease inhibitor cocktail), and thoroughly homogenized. Cell debris was removed via centrifugation, and the supernatant was collected. A second centrifugation step was performed to obtain a clear cell-free extract suitable for electrophoresis. Protein samples were subjected to SDS–PAGE, and gels were stained to visualize protein bands. Band intensities corresponding to the expected molecular weight of Tch36 were analyzed via densitometry using CLIQS 1D software (TotalLab, UK). Transformants showing strong, distinct Tch36-associated protein bands were selected for subsequent chitinase production and enzyme activity assays.

### Chitinase production and activity assay

Chitinase production was performed using recombinant *S. cerevisiae* strains expressing *tch36*. A single colony was inoculated into 5 mL uracil-deficient (ura⁻) medium and cultured for 48 h at 30 °C. An aliquot (500 μL) of this starter culture was transferred into 5 mL YEPD and incubated overnight. The secondary culture (5 mL) was subsequently inoculated into 40 mL modified SC production medium containing 6.7 g/L yeast nitrogen base, 2% (v/v) glycerol, 1.92 g/L yeast synthetic dropout supplement without uracil (Sigma-Aldrich, Cat. No. Y1501, USA), 50 mM potassium phosphate buffer (pH 6.0), and 5% (v/v) colloidal chitin. Cultures were grown for up to 5 days at 30 °C with shaking, after which supernatants were collected by centrifugation and used for all enzyme assays. Colloidal chitin was prepared from chitin powder (Sigma-Aldrich) using the hydrochloric acid–ethanol precipitation method (Deng et al. 2019; Subramanian et al. 2020), and the final pellet was resuspended in 50 mM potassium phosphate buffer (pH 6.0). The 3,5-dinitrosalicylic acid (DNS) reagent was prepared according to standard protocols (Božinović et al. 2023).

Chitinase activity was quantified by measuring the release of reducing sugars from colloidal chitin using a DNS assay. Briefly, 250 μL culture supernatant was mixed with 83 μL colloidal chitin substrate and incubated at 40 °C for 1 h. The reaction was stopped by centrifugation at 8000×*g* for 20 min, and 167 μL supernatant was combined with 167 μL DNS reagent and 10 μL 1% NaOH. After boiling for 5 min, cooling, and adding 333 μL sterile distilled water, absorbance was measured at 540 nm. Reducing sugar concentrations were determined from a standard curve generated using N-acetyl-D-glucosamine (GlcNAc). To express enzymatic activity in standard units, a standard curve was generated using commercial *Trichoderma viride* chitinase (Sigma-Aldrich, Cat. No. C8241) assayed under identical DNS conditions. One unit (1 U) of chitinase activity was operationally defined as the amount of enzyme required to release 1 μmol of GlcNAc-equivalent reducing sugars per hour at 40 °C. Absorbance values obtained from culture supernatants were converted to activity units (U/mL). Enzyme activity was expressed as U per mL of culture supernatant. All enzyme production experiments were performed using three independent biological replicates (*n* = 3), in which cultures were prepared on separate occasions. Chitinase activity assays were conducted in technical triplicate for each biological replicate, and the mean value obtained from each biological replicate was used as the statistical unit for analysis.

### Chitinase stability and colloidal chitin supplementation

Cell-free culture supernatants containing secreted chitinase were obtained by centrifugation of recombinant and mock S. cerevisiae cultures grown under the conditions described above and were used without further purification. For temperature-dependent storage stability, supernatants were aliquoted and stored at − 20 °C, 4 °C, 25 °C, or 40 °C for up to 9 days, and chitinase activity (U/mL) was measured at 0, 3, 6, and 9 days using the standard assay. For pH stability analysis, supernatants were adjusted to pH 3, 5, 7, or 9 using HCl or NaOH, incubated at room temperature, and assayed immediately (0 h) and after 6 h. To assess the effect of colloidal chitin supplementation, strains were cultured in SC–glycerol medium (2%, v/v glycerol) with or without 5% (w/v) colloidal chitin under identical cultivation conditions, and chitinase activity of culture supernatants was determined after 72 h; supernatant pH was measured at 0 h and 72 h using a calibrated pH meter. All experiments were performed using three independent biological replicates (n = 3), and statistical analysis was conducted by one-way ANOVA followed by Duncan’s multiple range test (*p* < 0.05).

### Antifungal activity assays

Recombinant *S. cerevisiae* strains were cultured in modified SC medium for 4 days at 30 °C. The cultures were centrifuged at 5000×*g* for 10 min to remove yeast cells, and the resulting supernatants were sterilized by filtration through a 0.45-µm membrane filter. The clarified, cell-free culture filtrate was used as the test agent in all antifungal assays. Culture filtrates were independently prepared for each biological replicate and harvested at identical incubation times with standardized OD600 values prior to supernatant collection.

For solid-medium assays evaluating colony growth rates and colony-forming unit (CFU) counts, the filtrate was mixed with an equal volume of 2 × potato dextrose agar (PDA) and poured into plates. Each plate was considered one technical replicate for statistical analysis. For liquid culture assays measuring mycelial dry weight, the filtrate was mixed with an equal volume of 2 × PDB, and each culture flask was treated as one technical replicate. Dose-dependent effects on hyphal growth were assessed by supplementing PDB with filtrate to final concentrations of 25% or 50% (v/v). For hyphal elongation assays, at least 50 individual germ tubes were randomly selected and measured per condition within each biological replicate (n ≥ 50 per biological replicate; n = 3), and the mean value from each biological replicate was used as the statistical unit.

Pathogenic fungi were precultured on PDA plates for 5 days at 30 °C. Conidia were harvested, filtered to remove hyphal fragments, and counted using a hemocytometer. The spore concentration was adjusted to ensure a uniform inoculum density across all experiments. All assays were performed using three independent biological replicates conducted on separate days (n = 3), each including five technical replicates per fungal strain and treatment condition.

### Protoplast formation assay

*A. nidulans* strains were grown on solid complete medium (CM) at 37 °C for 3 days. Conidia were harvested in 0.08% Tween 80, filtered through Miracloth to remove hyphal fragments, and adjusted to a concentration of 1 × 10^6^ conidia/mL. The conidial suspension was inoculated into liquid CM and incubated for 14 h at 37 °C with shaking (200 rpm) to generate mycelia. The resulting mycelia were collected by filtration and washed with sterile distilled water and osmotic buffer (0.6 M KCl, 10 mM NaCl). The washed mycelia were incubated for 2 h at 30 °C with gentle shaking (100 rpm) in either control buffer or experimental buffer. Three types of buffers were prepared for the assay. The control buffer contained 15 mg/mL VinoTaste Pro (Novozymes A/S, Denmark) and 8 mg/mL bovine serum albumin (BSA) in osmotic buffer. All treatments were conducted under identical protoplasting conditions to ensure standardized baseline cell wall–degrading activity across experimental groups, and was mixed 1:1 (v/v) with uninoculated liquid medium to match the nutrient composition of other treatments. The mock buffer included culture filtrate from a control *S. cerevisiae* strain carrying the empty vector. The experimental buffer was supplemented with culture supernatant from the recombinant M2-4 strain expressing *tch36*. All buffers were applied under identical conditions.

Protoplast formation was monitored by light microscopy throughout the incubation period. Following the reaction, protoplasts were recovered and washed according to a modified method of Pontecorvo et al. (1953), and the final yield was quantified using a hemocytometer.

### Statistical analysis

All experiments were performed using three independent biological replicates conducted on separate days (n = 3), unless otherwise specified. Data are presented as mean ± standard deviation (SD), and the mean value obtained from each biological replicate was used as the statistical unit for analysis. Statistical significance was evaluated using one-way analysis of variance (ANOVA) followed by Duncan’s multiple range test for comparisons involving more than two groups, performed using IBM SPSS Statistics for Windows (v29.0.2.0; IBM Corp., USA). For pairwise comparisons between two groups, an unpaired two-tailed Student’s *t*-test was conducted using jamovi (v2.6.26.0; The jamovi project, Australia). Differences were considered statistically significant at *p* < 0.05. When indicated in the figures, asterisks denote increasing levels of statistical significance (*p* < 0.05 (^*^), *p* < 0.01 (^**^), and *p* < 0.001 (^***^)).

In addition, the same statistical framework was applied to qRT-PCR data, with statistical analyses performed on *Δ*Ct values derived from biological replicates prior to fold-change calculation.

## Results and discussion

### Construction of a yeast-based heterologous secretion platform for *T. atroviride* chitinase Tch36

The *tch36* gene was successfully amplified as a 1035-bp cDNA fragment and inserted into pGEM-T Easy. Three recombinant clones with the correct insert size were obtained, and all were sequence-verified without any nucleotide substitutions. The validated sequence was deposited in GenBank under accession number OM240654. Sequence alignment revealed 98.0% nucleotide identity and 99.1% amino acid identity with the previously reported *chit36* sequence (GenBank accession KT992143), indicating that the Korean isolate encodes a highly conserved Tch36 homolog (Supplementary Table 2), consistent with previous functional characterization of *chit36* as an antifungal endochitinase in *Trichoderma* sp. (Viterbo et al. 2001), and recent polyphasic analyses highlighting the role of chitinase-related mechanisms in *T. atroviride* (González-Martínez et al. 2024).

To enable secretion in yeast, the rice α-amylase 1A signal peptide (Ramy1A) was fused to the 5′ end of the *tch36* coding region, generating full-length and mature variants that differed by the presence or absence of the native *T. atroviride* signal peptide. Chit36 was selected as a model enzyme because it is a secreted fungal endochitinase bearing a native signal peptide, as previously reported, allowing direct comparison between full-length and mature constructs in this system (Carsolio et al. 1994; Viterbo et al. 2001).

**Fig. 1:**
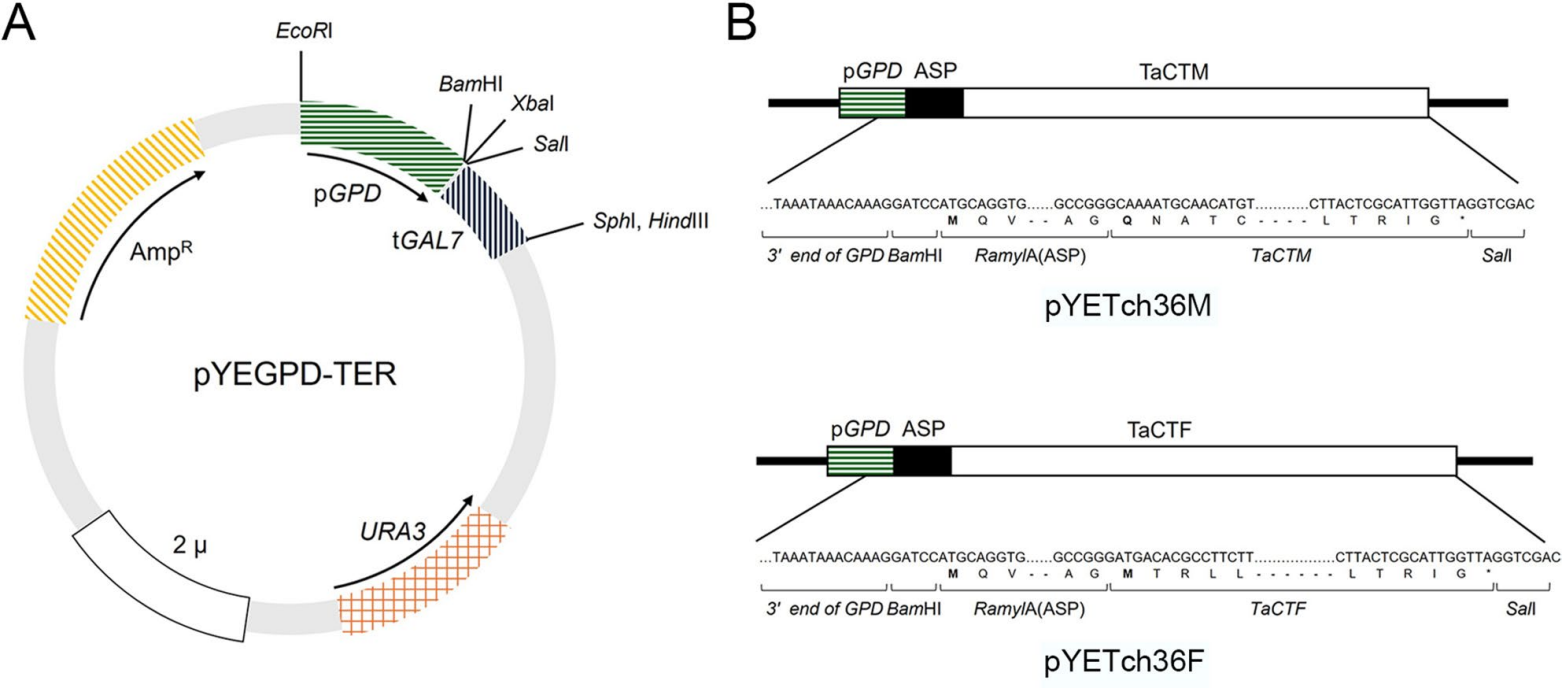
Schematic representation of the yeast expression vector used for Tch36 production. **A** Diagram of the pYEGPD-TER yeast expression vector, with boxed regions indicating genes or functional elements. **B** Structure of the recombinant construct inserted into the yeast expression vector, showing the organization of the glyceraldehyde-3-phosphate dehydrogenase (GPD) promoter, rice Amy1A signal peptide, and *tch36* coding sequence. The translation initiation codon and the first codon of Tch36 are highlighted in bold. Stop codons are indicated with asterisks. Abbreviations: pGPD, promoter of glyceraldehyde-3-phosphate dehydrogenase; ASP, rice Amy1A signal peptide; Tch36F, full-length Tch36 peptide; Tch36M, mature Tch36 peptide; tGAL7, terminator of galactose-1-phosphate uridylyltransferase. This construct design provides a structural framework for secretion of recombinant Tch36 and enables comparison between full-length and mature peptide configurations

The resulting fusion fragments, consisting of the Ramy1A signal peptide and the *tch36* coding region (1145 bp and 1070 bp, respectively), were assembled into the episomal vector pYEGPD-TER under the constitutive GPD promoter (Fig. 1A), and three clones were recovered for each construct, all carrying intact inserts without substitutions, insertions, or deletions (Fig. 1B). Collectively, these results demonstrate the successful construction of a heterologous secretion platform in *Saccharomyces cerevisiae* that employs a plant-derived signal peptide to facilitate the extracellular delivery of a fungal chitinase.

The generation of both full-length and mature *tch36* variants was intended to evaluate the functional contribution of the native 25-amino-acid signal peptide during heterologous secretion in yeast. This design enables the system to compare secretion behavior associated with the presence or absence of the endogenous fungal signal sequence relative to the Ramy1A secretion signal. This secretion strategy is consistent with previous reports demonstrating the broad utility of the Ramy1A signal peptide in enhancing heterologous protein secretion in yeast (Shin et al. 2004; Lee et al. 2006; Heo et al. 2007; Kim et al. 2010; Nguyen et al. 2015; So et al. 2025) and supports the view that optimization of N-terminal signal regions can markedly improve secretory efficiency in yeast expression systems (Idiris et al. 2010). The successful assembly of both constructs establishes the modular framework of this secretion platform, supporting its applicability for the extracellular expression of diverse fungal enzymes in yeast hosts.

### Quantification of *tch36* expression and temporal transcript profiles

Transformation of *S. cerevisiae* Y2805 with pYETch36F and pYETch36M yielded more than 100 uracil-prototrophic colonies for each construct. Colony PCR screening of randomly selected transformants consistently produced the expected 1,035-bp *tch36* amplicon, confirming correct plasmid introduction (Supplementary Fig. S1). The validated transformants were designated TYETch-F (full-length) and TYETch-M (mature).

Quantitative transcript profiling revealed considerable variability among individual transformants, as well as distinct differences between the full-length and mature constructs. All transformants exhibited markedly elevated *tch36* transcript levels relative to the empty-vector control (mock). Among the TYETch-M strains, M2-4 expressed the highest transcript level (~ 3.6 × 10^4^-fold above mock), whereas the remaining clones ranged from ~ 2.0 × 10^4^ to 3.2 × 10^4^ (Supplementary Fig. S2A). TYETch-F strains displayed lower, yet still robust, expression levels, ranging from ~ 1.4 × 10^4^ to 2.1 × 10^4^-fold (Supplementary Fig. S2B). These patterns are consistent with previously reported effects of signal peptide configuration on protein processing and secretion; however, they do not establish a direct effect on transcriptional efficiency (Idiris et al. 2010; Gasser and Mattanovich 2018; Yu et al. 2013). Based on these results, four high-expressing transformants − TYETch-M (M2-1 and M2-4) and TYETch-F (F3-1 and F5-2) − were selected for temporal transcript analysis.

**Fig. 2:**
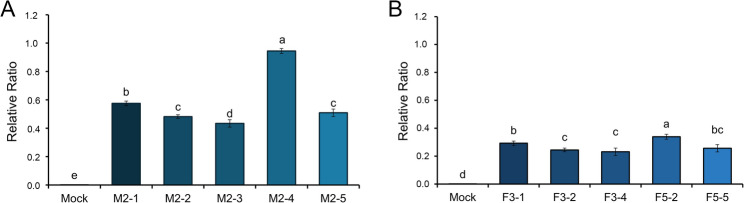
Temporal expression pattern of the *tch36* gene in recombinant *S. cerevisiae*. Quantitative real-time polymerase chain reaction (qRT-PCR) was performed to evaluate the temporal expression of the *tch36* gene in recombinant *S. cerevisiae* transformants. A single representative transformant was selected from each strain: M2-1 for TYETch-M (**A**) and F5-2 for TYETch-F (**B**). Total RNA was extracted from 3-, 4-, and 5-day-old cultures. Gene expression levels were normalized relative to those of the endogenous housekeeping *GPD* gene. Light gray bars represent the mock strain (vector only), labeled as “Mock” in the figure, and colored bars represent the selected recombinant strains. Error bars represent SD from three independent biological replicates (*n* = 3). Statistical significance was determined using one-way ANOVA followed by Duncan’s multiple range test (*p* < 0.05). These results indicate successful transcription of the heterologous *tch36* gene in recombinant yeast relative to the mock control

To capture dynamic changes in expression during cultivation, *tch36* transcript levels were monitored from days 2 to 5 (Fig. 2). As expected, the mock strain maintained only the baseline signal throughout, validating assay specificity. The TYETch-M strains exhibited early and robust activation, with transcript levels exceeding ~ 2 × 10^5^-fold relative to the mock strain on day 2. Both M2-1 and M2-4 showed a modest increase on day 4 compared with day 3. Across all corresponding time points, the TYETch-M strains consistently maintained approximately 3- to 15-fold higher transcript abundance compared to the TYETch-F strains. Although expression declined on day 5, overall transcript levels remained higher in the mature construct under the tested conditions. In contrast, the TYETch-F strains followed the transcriptional kinetics typical of *S. cerevisiae*, characterized by moderate induction on day 2, a clear peak on day 3 (~ 10^4^-fold), and strain-dependent fluctuations on day 4, followed by reduced expression on day 5. These observations indicated construct-associated differences under the tested conditions. However, these data do not establish mitigation of a defined transcriptional or secretory bottleneck (Idiris et al. 2010; Delic et al. 2013).

Together, these temporal expression profiles indicate a clear functional divergence between the two constructs: TYETch-M strains achieve rapid and reinforced transcription, while TYETch-F strains follow a more typical mid-phase peaking pattern. These insights provide a strong mechanistic basis for subsequent analyses assessing whether elevated transcript abundance correlates with enhanced Tch36 production and extracellular enzyme activity under identical cultivation conditions.

### Functional secretion of Tch36 and medium optimization

**Fig. 3:**
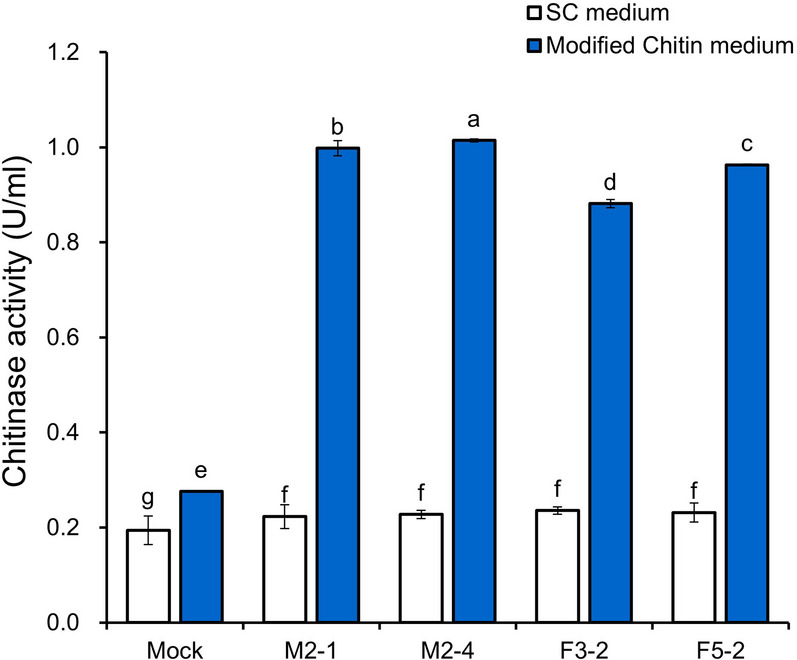
Effects of chitinase activity in modified chitin medium. Recombinant *S. cerevisiae* transformants and a mock strain were cultured in synthetic complete (ura^−^) medium or modified chitin media for 4 days. Chitinase activity in the culture filtrates was measured after incubation. The recombinant transformants included TYETch-M (M2-1 and M2-4) and TYETch-F (F3-2 and F5-2). The mock strain (Mock) carried the empty episomal plasmid vector. White and blue bars in the figure represent SC (ura^−^) medium and modified chitin medium, respectively. Error bars represent SD from three independent biological replicates (n = 3). Statistical significance was determined using one-way ANOVA followed by Duncan’s multiple range test (*p* < 0.05). Recombinant strains cultured in modified chitin medium exhibit higher extracellular chitinase activity compared with the mock strain

The functional secretion and extracellular catalytic activity of Tch36 were first evaluated by measuring chitinase activity in culture supernatants after 3 days of cultivation in standard SC medium and in a modified SC (MSC) medium supplemented with glycerol and colloidal chitin (Fig. 3). Chitinase activity values are expressed as U per mL of culture supernatant as defined in the Materials and Methods. The recombinant strains analyzed included TYETch-M (M2-1 and M2-4), TYETch-F (F3-1 and F5-2), and the empty-vector control (mock). In standard SC medium, all recombinant strains exhibited a consistent increase in extracellular chitinase activity of approximately 1.1–1.2-fold relative to the mock control, indicating functional secretion of Tch36 under basal cultivation conditions. Under these conditions, no statistically significant difference was observed between the full-length and mature constructs. It should be noted that the mock strain displayed low but detectable basal chitinase activity, which is attributable to endogenous yeast chitinases primarily involved in cell wall remodeling during cell division rather than extracellular chitin degradation (Kuranda and Robbins 1991; Cabib et al. 2001). Together, these observations indicate that constitutive GPD-driven expression is sufficient to support detectable extracellular chitinase activity in standard SC medium; however, higher-level extracellular accumulation of catalytically active Tch36 requires optimization of medium composition and cultivation conditions.

By comparison, extracellular chitinase activity substantially increased in MSC medium. The TYETch-M strains (M2-1 and M2-4) showed 3.6- and 3.7-fold increases, respectively, whereas the TYETch-F strains (F3-1 and F5-2) exhibited 3.2- to 3.5-fold increases relative to the mock control. To distinguish the individual contributions of medium components, chitinase activity was additionally measured in SC–glycerol medium without colloidal chitin supplementation (Supplementary Fig. 4A). Under glycerol-only conditions, extracellular chitinase activity remained at basal levels and did not differ substantially from the SC control, indicating that glycerol alone did not account for the enhanced activity observed in MSC medium. In contrast, supplementation with colloidal chitin resulted in a marked increase in extracellular chitinase activity, supporting a substrate-associated effect on enzyme accumulation and/or stability. Because the mock strain displayed only basal chitinase activity intrinsic to *S. cerevisiae* under identical conditions, the enhanced extracellular activity observed in recombinant strains can be attributed primarily to the secretion of heterologous Tch36 rather than to endogenous yeast chitinases, which are primarily associated with cell wall remodeling and are not secreted at levels sufficient for extracellular substrate degradation. Furthermore, because *tch36* transcription was constitutively driven by the GPD promoter, the elevated activity observed in MSC medium is unlikely to be explained solely by transcriptional induction. Instead, these data indicate that medium composition positively influences post-transcriptional processes, including secretion efficiency, protein folding, and/or extracellular stability of Tch36. This interpretation is consistent with previous reports demonstrating that glycerol alleviates carbon catabolite repression and enhances heterologous protein secretion in yeast (Gancedo 1998; Cereghino and Cregg 2000; Ahmad et al. 2014), and that chitinous substrates can promote the accumulation or stabilization of extracellular chitinases by reducing proteolytic degradation or enhancing enzyme–substrate interactions (Wang et al. 2020a, b; Subramanian et al. 2020). Notably, this medium-dependent enhancement suggests that optimization of extracellular enzyme performance in yeast can be achieved without further genetic modification, by strategically modulating cultivation parameters that favor secretion and enzyme persistence.

**Fig. 4:**
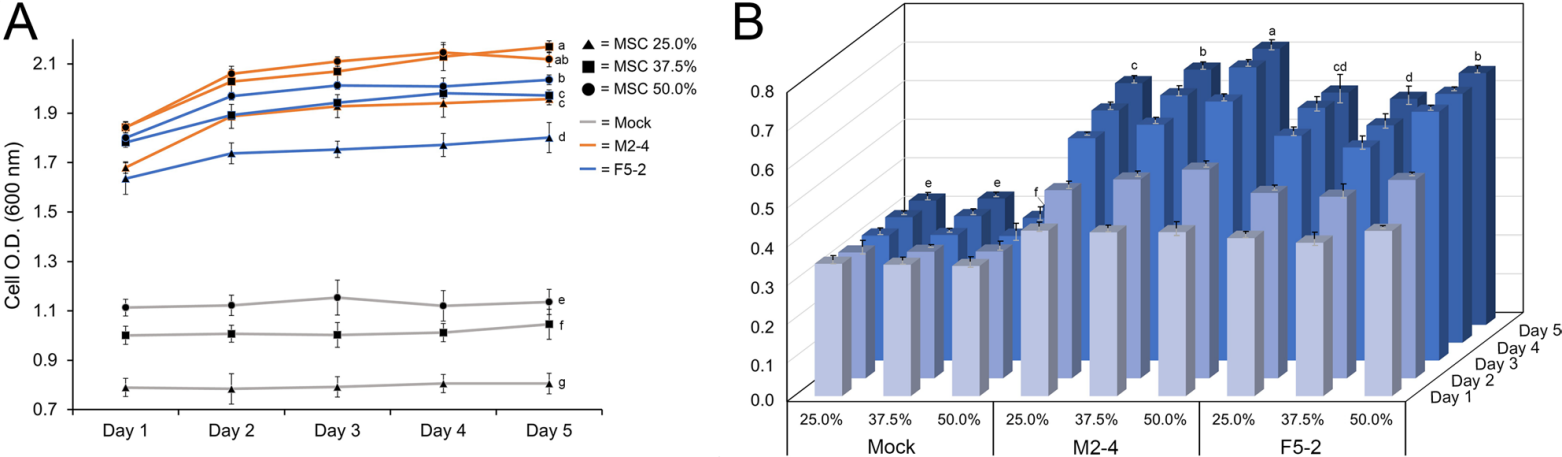
Growth and chitinase activity of recombinant *S. cerevisiae* in response to different concentrations of colloidal chitin. **A** Cell growth of recombinant *S. cerevisiae* transformants cultured in modified chitin medium containing 25.0%, 37.5%, or 50.0% colloidal chitin was measured daily for 5 days *by OD*_*600*_*.* **B** Chitinase activity was assessed from culture supernatants collected daily under the same conditions. All strains were cultured under identical conditions. Recombinant transformants included TYETch-M (M2-4) and TYETch-F (F5-2). The mock strain (Mock) carried the empty episomal plasmid vector. Error bars represent SD from three independent biological replicates (n = 3). Statistical significance was determined using one-way ANOVA followed by Duncan’s multiple range test (*p* < 0.05). Variation in colloidal chitin concentration is associated with changes in yeast growth dynamics and secreted chitinase activity over time

To further examine the influence of substrate load and cultivation duration, representative high-expressing strains M2-4 (TYETch-M) and F5-2 (TYETch-F) were cultivated for up to 5 days in MSC medium supplemented with increasing concentrations of colloidal chitin (final, v/v of colloidal chitin slurry: 25%, 37.5%, and 50%) (Fig. 4). These slurry volumes corresponded to approximate final colloidal chitin concentrations of 2%, 3%, and 5% (w/v), respectively. Extracellular chitinase activity increased progressively across this concentration range, with the highest activity observed at 5%, which was therefore selected for subsequent experiments within the tested laboratory-scale conditions. Both recombinant strains exhibited rapid growth through day 3, reaching OD₆₀₀ values of 2.3–2.6 before entering a stationary phase, whereas the mock strain remained below OD₆₀₀ 1.1 and did not show a clear transition to stationary growth. Importantly, no detectable growth inhibition was observed in recombinant strains even at the highest substrate concentration, indicating that chitin supplementation did not impose a measurable physiological burden on strains secreting Tch36.

In contrast, the limited growth of the mock control under chitin-rich conditions suggests that endogenous yeast chitinase activity is insufficient to confer a comparable growth advantage, whereas secretion of Tch36 is associated with sustained growth performance in the same medium. This differential growth behavior suggests that extracellular Tch36 may be associated with improved growth performance of recombinant strains under substrate-rich conditions; however, the extent to which colloidal chitin directly contributes to yeast carbon metabolism was not determined in this study. Extracellular chitinase activity exhibited an overall gradual increase with increasing cultivation time and substrate concentration. The most pronounced increase was observed between days 2 and 3 of cultivation, whereas from day 3 through day 5 the activity either plateaued or increased more gradually. Across all substrate concentrations and time points, strain M2-4 consistently displayed slightly higher extracellular chitinase activity than strain F5-2. This activity pattern suggests construct-dependent variation under the tested conditions but does not demonstrate that removal of the native signal peptide directly improves compatibility with the yeast secretory pathway.

Notably, the temporal profiles of *tch36* transcription and extracellular chitinase activity did not fully coincide. TYETch-M strains exhibited high transcript abundance on day 2 followed by a gradual decline, whereas TYETch-F strains reached maximal transcript levels on day 3 before decreasing toward day 5 (Fig. 2). Despite these differences in transcriptional dynamics and the overall decline in mRNA abundance at later time points, extracellular chitinase activity remained relatively stable from days 3 to 5. Such partial decoupling between transcript levels and extracellular enzyme output is a well-recognized feature of yeast expression systems, in which extracellular protein accumulation is governed not only by transcriptional activity but also by downstream processes, including secretion kinetics, extracellular residence time, and protein turnover (Delic et al. 2013; Csárdi et al. 2015; Liu et al. 2016). Accordingly, the sustained extracellular activity observed in this study likely reflects the continued presence of previously secreted Tch36 in the cultivation medium rather than ongoing transcriptional input alone. These results underscore that effective extracellular enzyme performance in yeast cannot be inferred solely from transcript abundance and instead depends on the integrated efficiency of secretion and extracellular persistence, emphasizing the importance of optimizing cultivation conditions to maximize functional enzyme accumulation.

**Fig. 5:**
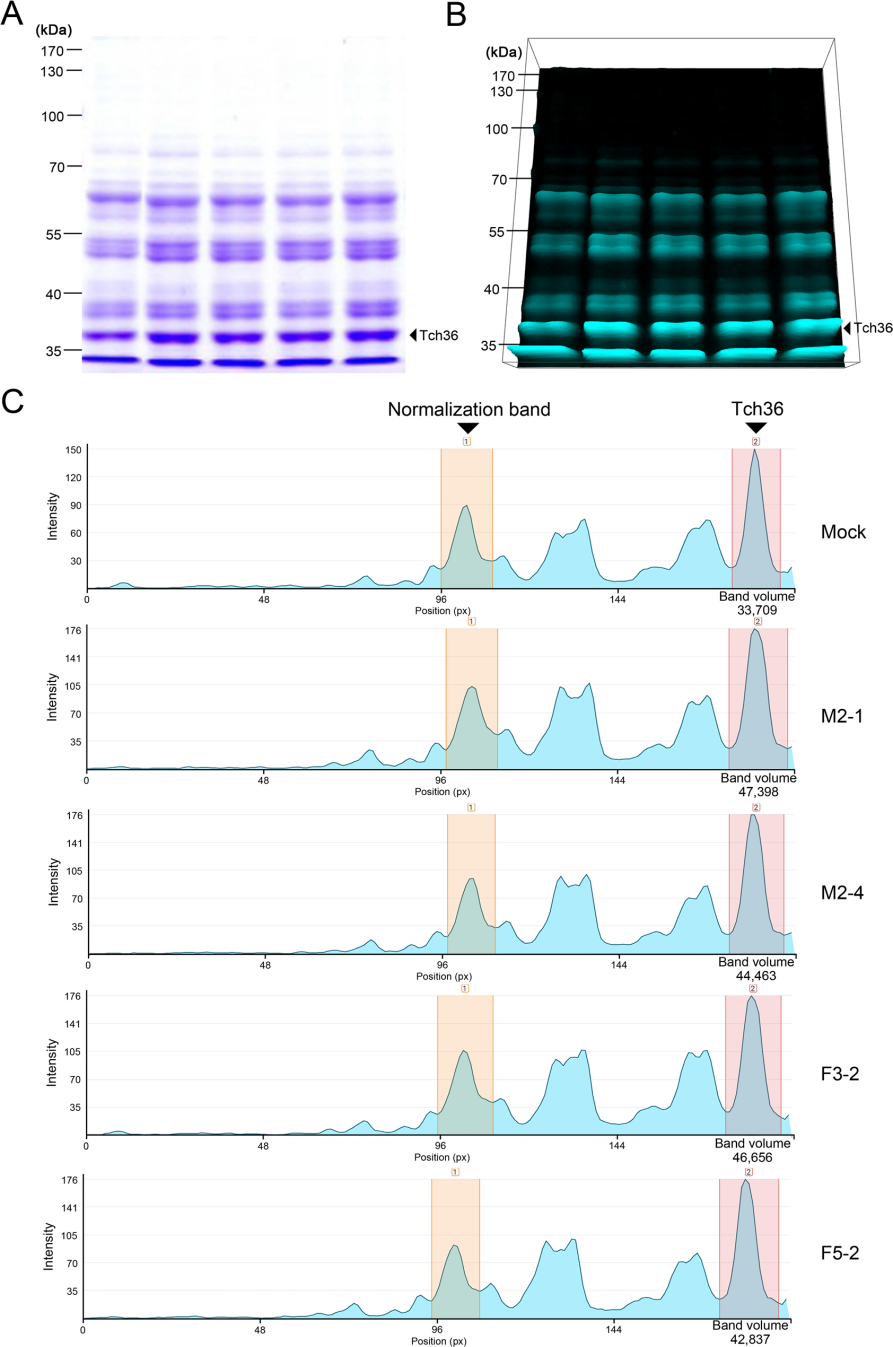
Analysis of recombinant Tch36 protein accumulation in *S. cerevisiae* by 1D-PAGE and densitometry. **A** Coomassie brilliant blue–stained SDS-PAGE showing total protein profiles of *S. cerevisiae* transformants producing recombinant Tch36 and the mock control strain. The transformants analyzed include two independent TYETch-M strains (M2-1 and M2-4) and two independent TYETch-F strains (F3-2 and F5-2), as indicated by the lane labels. Protein bands migrating at the expected molecular size of Tch36 are indicated. **B** Three-dimensional intensity rendering of the same 1D-PAGE gel generated using Phoretix 1D software, visualizing relative differences in protein band intensity across lanes. **C** Densitometric analysis of selected protein bands. The normalization band and the putative Tch36 band are annotated for each sample, and the corresponding peak intensities and band volumes are shown. The presence of a protein band at the expected molecular size, together with increased band intensity in transformants, is consistent with accumulation of recombinant Tch36

To support that the observed extracellular activity originated from recombinant Tch36, intracellular protein accumulation was examined prior to secretion assays by SDS–PAGE followed by densitometric analysis (Fig. 5). Protein bands migrating at the expected molecular mass of Tch36 (~ 36.4 kDa) were consistently detected in recombinant strains with higher relative intensities than in the mock control, supporting intracellular production of recombinant Tch36 before secretion and extracellular activity measurements.

To further evaluate the stability profile of secreted Tch36, temperature-dependent storage stability and pH-dependent activity were assessed (Supplementary Figs. 3 and 4). Under frozen storage (− 20 °C), chitinase activity remained stable over 9 days (Supplementary Fig. 3A), whereas modest time-dependent reductions were observed at 4 °C and 25 °C (Supplementary Fig. 3B–C). In contrast, storage at 40 °C resulted in a progressive decrease in residual activity (Supplementary Fig. 3D). The mock strain maintained consistently low activity across all temperature conditions.

Culture supernatant pH after 72 h cultivation remained within a narrow acidic range across all tested media (Supplementary Fig. 4B), indicating that enhanced extracellular activity was not attributable to bulk pH differences. When evaluated under defined pH conditions, secreted Tch36 maintained activity within mildly acidic to neutral ranges (Supplementary Fig. 4C–D), while moderate reductions were observed under alkaline conditions and partial strain-dependent sensitivity under strongly acidic conditions. Collectively, these results demonstrate that secreted Tch36 exhibits stable activity under low-temperature storage and physiologically relevant pH conditions, with predictable sensitivity to elevated temperature and extreme pH environments.

Collectively, these results demonstrate that both full-length and mature constructs are capable of secreting catalytically active Tch36, while the magnitude of extracellular enzyme output is strongly influenced by cultivation conditions, particularly medium composition and substrate availability. Under MSC conditions, the mature construct (TYETch-M) tended to yield higher extracellular activity at elevated chitin loads, whereas under basal or lower-substrate conditions the full-length construct (TYETch-F) exhibited comparable performance. These trends indicate that differences between construct architectures become more apparent under substrate-rich conditions, rather than being uniformly expressed across all cultivation environments. Together, these findings establish a functional linkage between construct architecture, cultivation environment, and extracellular enzyme output, and highlight medium optimization as a critical parameter for maximizing the performance of yeast-based chitinase production platforms.

### Growth inhibition of phytopathogenic fungi

**Fig. 6:**
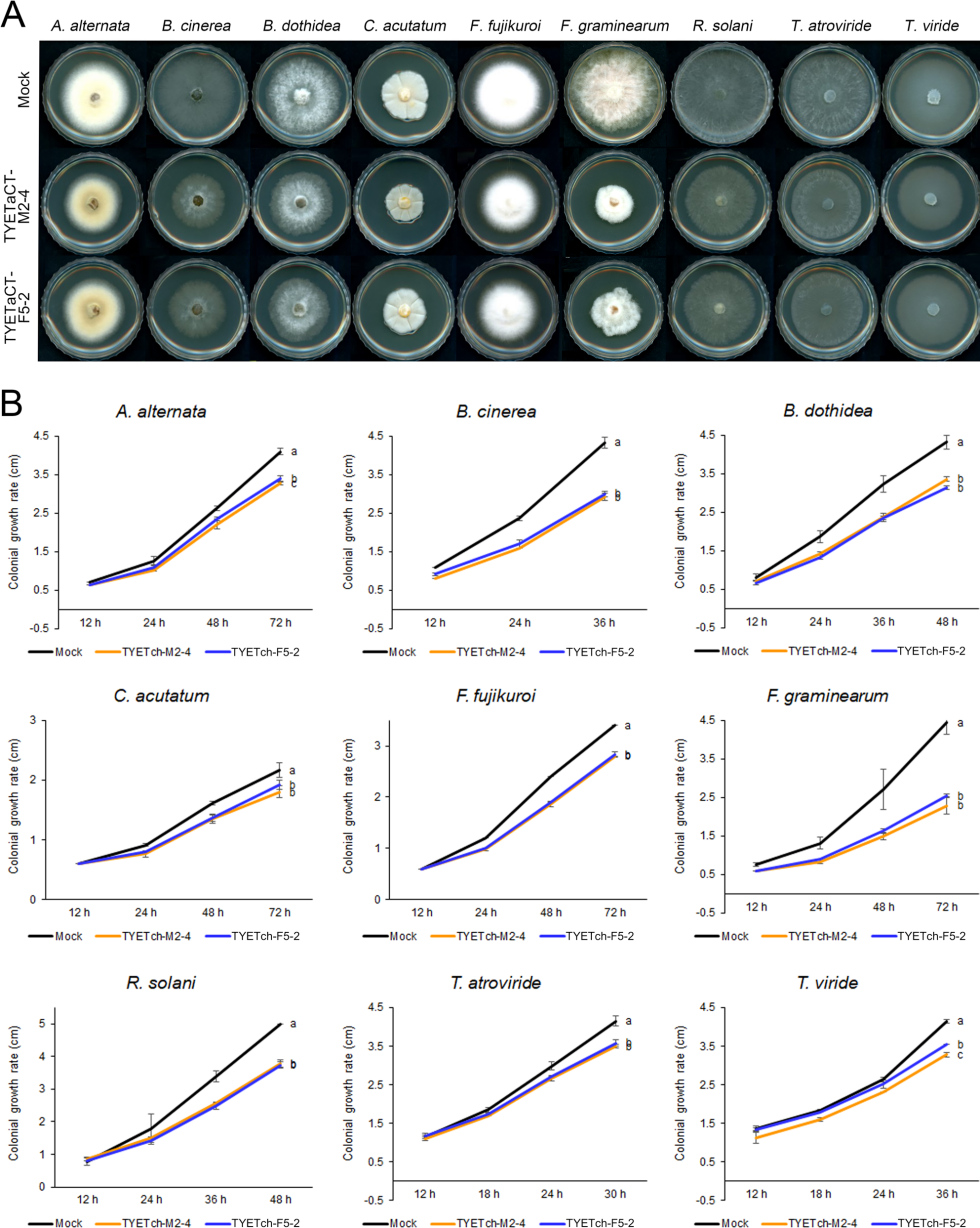
Antifungal activity of recombinant chitinase-containing culture filtrates against nine phytopathogenic fungi. **A** Colony morphology of nine phytopathogenic fungal strains after 48 h of incubation on potato dextrose agar (PDA) plates supplemented with culture filtrates from recombinant *S. cerevisiae* strains expressing chitinase. Mycelial agar blocks, prepared using a cork borer (No. 3), were placed in the center of each plate (center-point inoculation). **B** Quantitative analysis of fungal growth inhibition was performed by measuring colony diameters at defined time intervals (every 6 or 12 h, depending on the growth rate of each strain). Recombinant culture filtrates were derived from the TYETch-M and TYETch-F strains, and mock culture filtrate (Mock) containing only the empty vector was used as a control. Error bars represent standard deviations from three independent biological replicates conducted on separate days (*n* = 3), each including three technical replicate plates per fungal strain and treatment condition. Statistical significance was determined by one-way ANOVA followed by Duncan’s multiple range test (*p* < 0.05). Recombinant Tch36-containing culture filtrates are associated with reduced radial growth of diverse phytopathogenic fungi compared with the mock control

Secreted Tch36 exhibited clear and statistically significant antifungal effects against all nine phytopathogenic fungi tested. Representative colony morphologies under each treatment and quantitative changes in colony diameters over time are presented in Fig. 6. In every species examined, colony expansion was visibly reduced in plates containing culture supernatants from M2-4 (TYETch-M) and F5-2 (TYETch-F), whereas the mock supernatant supported full-colony expansion.

To allow direct comparison among fungal species with different intrinsic growth rates, the sizes of mock-derived colonies were normalized to 100%, and relative growth under Tch36-containing supernatants was expressed as a percentage of this baseline (Supplementary Table 3). Both recombinant strains significantly suppressed fungal growth compared to the normalized mock values (*p* < 0.05). Among the pathogens tested, *F. graminearum* was particularly sensitive, exhibiting relative growth of 51.59% ± 4.96% under M2-4 and 57.14% ± 1.19% under F5-2. Likewise, *B. cinerea* showed substantial growth reduction (67.44% ± 2.13% and 69.30% ± 1.61%, respectively). Across most species, M2-4 tended to exhibit stronger inhibitory effects than F5-2. This trend is consistent with the higher extracellular chitinase activity observed for the mature construct, supporting a functional contribution of construct architecture to antifungal performance under the conditions tested.

A key interpretive point is that the observed inhibition cannot be attributed to chitin or other medium components. Because all values were normalized to the mock supernatant (derived from an empty-vector *S. cerevisiae* transformant without a *tch36* insert but with the same basal endogenous chitinase background and medium constituents) any minor effects originating from intrinsic components were inherently controlled for. Therefore, the additional inhibition observed with M2-4 and F5-2 directly reflected Tch36-specific antifungal activity, effectively addressing a common concern that substrate-derived artifacts or background enzyme activities might confound interpretation.

The variability in inhibition across fungal species likely reflects intrinsic differences in chitin accessibility, cell wall rigidity, and vulnerability to enzymatic degradation, factors that can influence chitinase sensitivity (Benítez et al. 2004; Swiontek Brzezinska et al. 2014; Harman 2006; Wang et al. 2020a, b; Subramanian et al. 2020; Abdelraouf et al. 2024; Fisher et al. 2020). Importantly, the broad-spectrum activity observed here was achieved even with diluted culture supernatants, highlighting the functional robustness of Tch36 as a biocontrol-active enzyme.

Together, the results shown in Fig. 6 and Supplementary Table 3 demonstrate that *S. cerevisiae*-secreted Tch36 suppresses colony expansion across multiple agriculturally significant phytopathogens. The consistently stronger inhibition observed for the mature-construct strain M2-4 was associated with its higher transcript levels and extracellular activity under the tested conditions. However, this association does not establish a definitive mechanistic advantage attributable solely to signal peptide removal. The breadth of antifungal activity observed, spanning fungi with distinct ecological niches and cell-wall compositions, is consistent with established models of chitinase-mediated antagonism, in which enzymatic disruption of chitin-linked structural components compromises hyphal integrity and colony establishment (Benítez et al. 2004; Harman 2006; Wang et al. 2020a, b; Subramanian et al. 2020). Importantly, these inhibitory effects were achieved using crude, unpurified culture supernatants, highlighting the operational simplicity and scalability of a yeast-based secretion platform. Given the safety, industrial relevance, and regulatory familiarity of *S. cerevisiae*, these findings support the practical potential of Tch36-producing strains (or their culture filtrates) as deployable antifungal resources in agricultural biotechnology and bioprocess applications.

### Growth inhibition of opportunistic animal-pathogenic *Aspergillus* species

**Fig. 7:**
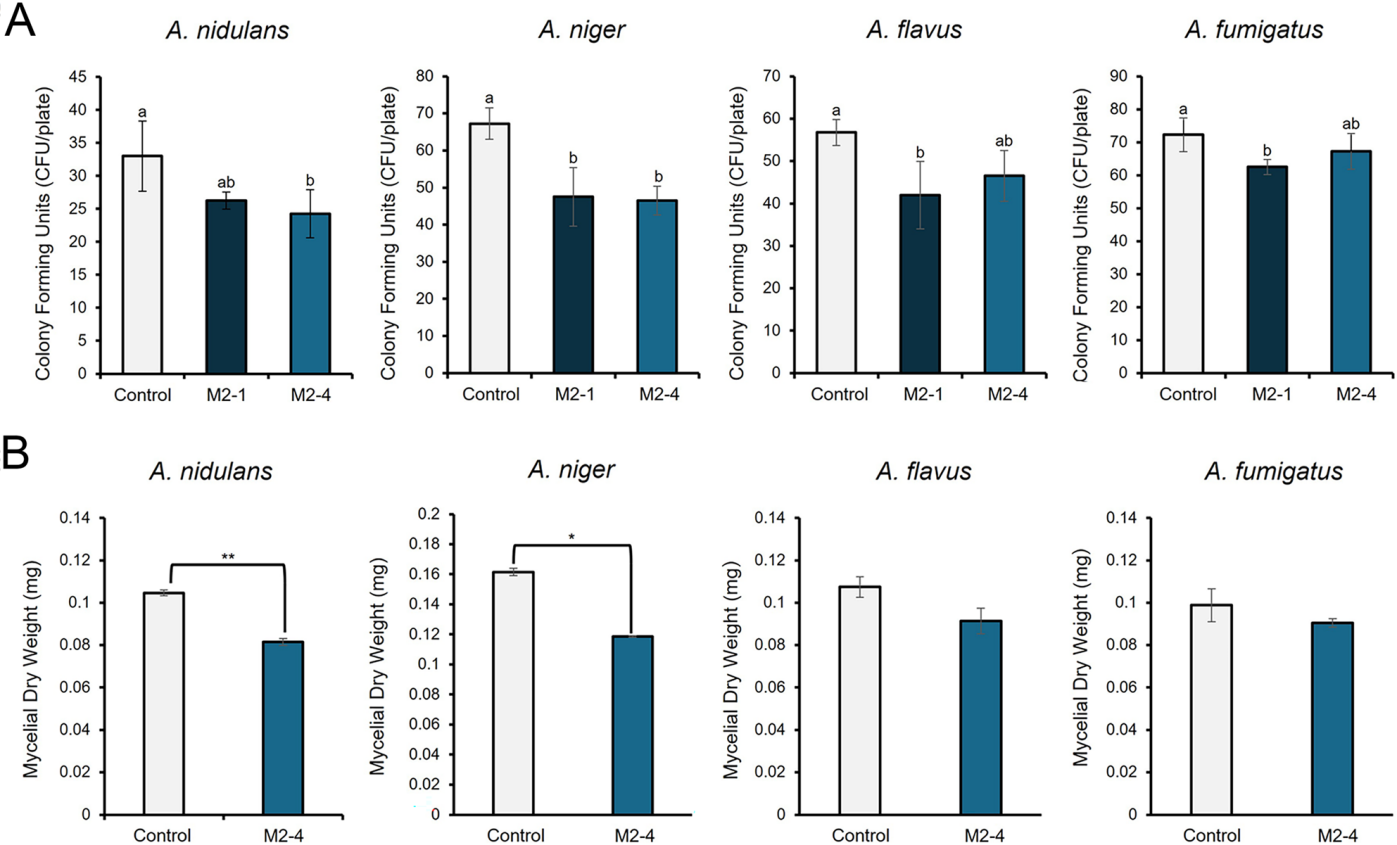
Antifungal activity of chitinase-containing media against animal pathogenic fungi. **A** Survival of asexual spores of *A. niger*, *A. flavus*, and *A. fumigatus* on potato dextrose agar-based solid media supplemented with recombinant *S. cerevisiae* culture filtrates. Asexual spores (100 per plate) were spread onto the media, and colony forming units (CFUs) were counted after incubation. *A. nidulans* was included as a reference control. **B** Fungal biomass production was measured in liquid potato dextrose broth medium containing 50% (v/v) recombinant culture filtrate. Conidia (10^6^ spores/mL) of the same fungal species were cultured for 24 h, and dry weights of the harvested mycelial pellets were recorded. The recombinant culture filtrates were obtained from *S. cerevisiae* strains TYETch-M (M2-4) expressing the *tch36* gene. A mock strain carrying the empty vector was used as a negative control (Mock). Error bars represent SD from three independent biological replicates conducted on separate days (n = 3). Each biological replicate included five technical replicates per condition. For panel A, statistical significance was determined using one-way ANOVA followed by Duncan’s multiple range test (different letters indicate significant differences, *p* < 0.05). For panel B, pairwise comparisons between Control and M2-4 were performed using Student’s *t*-test (*p* < 0.05; *p* < 0.01, as indicated by asterisks). Reduced CFU recovery and decreased fungal biomass are consistent with antifungal effects of recombinant Tch36 against opportunistic *Aspergillus* species

To assess whether secreted Tch36 also has activity against opportunistic fungal species associated with animals, including humans, culture supernatants from the mature-construct transformants were evaluated against four *Aspergillus* strains: *A. niger*, *A. nidulans* FGSC A4, *A. flavus*, and *A. fumigatus*. In a spore viability assay (Fig. 7A), both M2-1 and M2-4 significantly reduced CFU formation compared to the mock-treated control. Specifically, CFU counts decreased by 30.9% in *A. niger*, 26.4% in *A. nidulans*, 26.0% in *A. flavus*, and 13.5% in *A. fumigatus*, and all reductions were statistically significant (p < 0.05). These results indicate that Tch36 suppresses spore viability across these species. Hyphal growth assays further supported this inhibitory trend (Fig. 7B). When culture supernatant from the highest-expressing strain (M2-4) was applied in liquid culture, statistically significant reductions in mycelial dry weight were observed for *A. niger* (26.7%) and *A. nidulans* (22.1%). Although *A. flavus* and *A. fumigatus* also exhibited decreases in biomass, the changes did not reach statistical significance. The use of both CFU-based viability measurements (Fig. 7A) and biomass-based hyphal growth assays (Fig. 7B) provides complementary evidence, each capturing a distinct and biologically relevant stage of fungal development. Together, these datasets support the conclusion that secreted Tch36 disrupts multiple dimensions of fungal growth.

Importantly, the mock-treated cultures consistently exhibited the highest CFU counts and biomass levels, demonstrating that the inhibitory effects observed in the Tch36-treated samples cannot be attributed to medium components or background activities. Because all values were normalized to the mock supernatant (derived from an empty-vector strain exhibiting only minimal baseline activity) any minor effects associated with intrinsic medium constituents were inherently controlled for. Thus, the additional suppression observed in Tch36-containing samples represents a Tch36-specific antifungal effect, consistent with previous reports of chitinase-mediated interference with pathogenic *Aspergillus* species (Deng et al. 2019; Abdelraouf et al. 2024).

Collectively, these results demonstrate that yeast-secreted Tch36 exhibits clear and consistent antifungal activity not only against plant pathogens but also against opportunistic fungal species of relevance to animal (including human) health. Notably, these inhibitory effects were achieved using crude, unpurified culture supernatants, underscoring the applied potential of the *S. cerevisiae* secretion platform for bioprocessing and antifungal biotechnology.

### Early-stage inhibition of spore germination and initial hyphal growth

To provide further mechanistic insights into how Tch36 impairs fungal development, we examined whether its inhibitory effects extend to the earliest stages of fungal growth. Two representative fungi, *Botrytis cinerea* and *Aspergillus niger*, were selected because they exhibited pronounced growth suppression. Evaluating both species allowed us to determine whether early developmental inhibition is a generalizable feature across taxonomically distinct fungal groups.

**Fig. 8:**
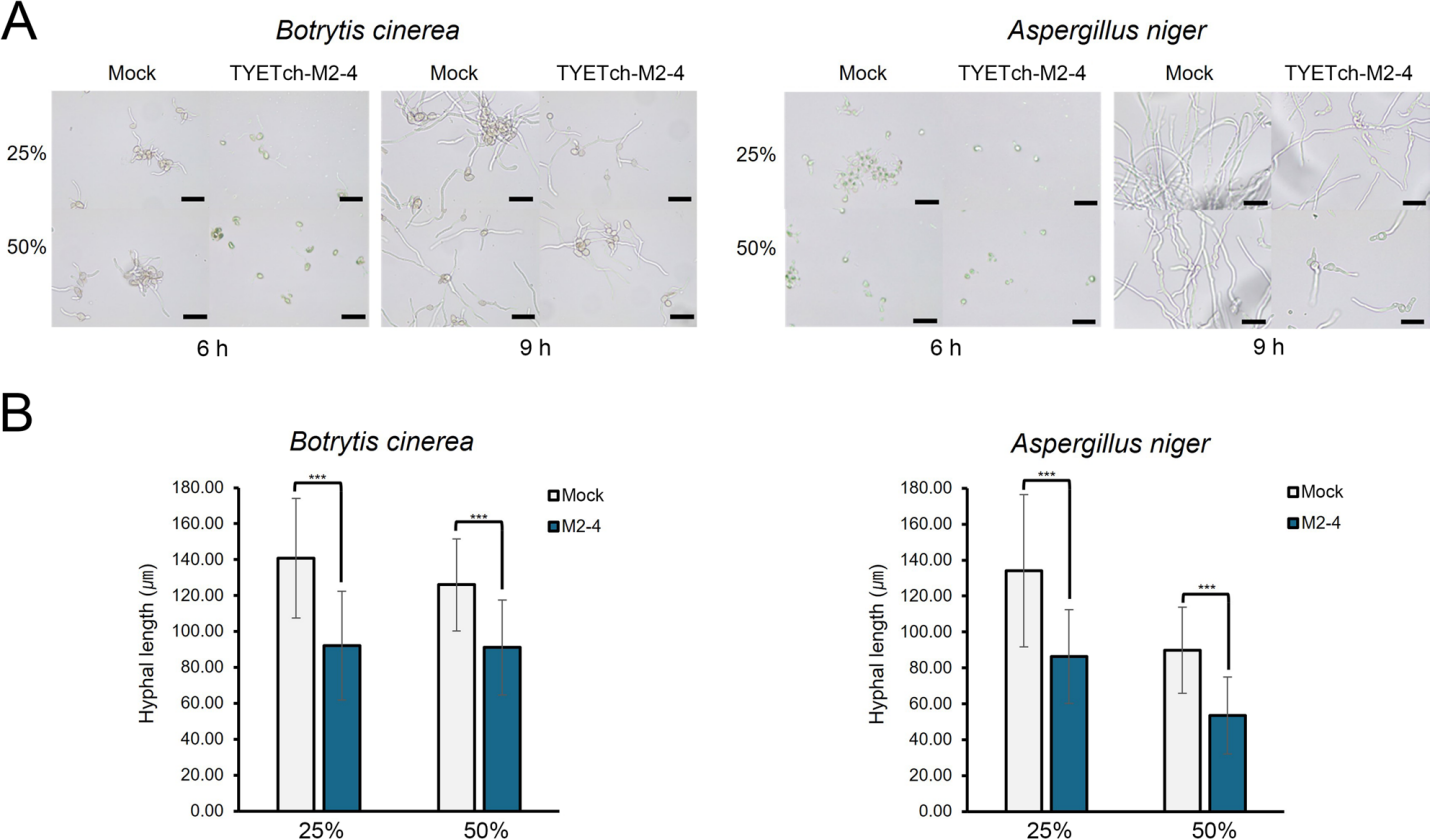
Antifungal effects of recombinant chitinase on conidial germination and early hyphal growth of *B. cinerea* and *A. niger*. **A** Light microscopy observation of conidial germination and hyphal development in *B. cinerea* and *A. niger* cultured in potato dextrose broth-based medium supplemented with 25% or 50% (v/v) recombinant culture filtrates (TYETch-M). Morphological changes were monitored at 6 and 9 h after inoculation. Scale bar: 50 μm. **B** Hyphal lengths were measured quantitatively at 9 h using an integrated ocular micrometer to evaluate the inhibitory effects of each treatment. For each biological replicate, at least 50 individual hyphae were randomly selected and measured per condition (*n* ≥ 50 per biological replicate; *n* = 3). Inhibition rates were calculated based on the mean hyphal length of each biological replicate. Error bars represent the standard deviation (SD) of three independent biological replicates. Statistical significance between Mock and M2-4 within each concentration was determined using Student’s t-test. ***p < 0.001. Recombinant filtrate is associated with delayed conidial germination and reduced early hyphal elongation in a concentration-dependent manner

Spores were exposed to 25% or 50% (v/v) culture filtrates from the mature-construct strain M2-4, and germination dynamics were monitored up to 9 h (Fig. 8A). In mock-treated controls, both fungi readily germinated, producing elongated germ tubes by 6–9 h. In contrast, spores treated with Tch36-containing filtrates displayed markedly reduced germination frequencies and pronounced defects in germ-tube elongation. At 50% filtrate, many spores either failed to germinate or produced only short, abortive hyphae, indicating substantial disruption of early developmental progression. Quantitative analysis of early hyphal elongation was performed at 9 h by measuring germ-tube lengths using light microscopy (≥ 50 per condition across three independent biological replicates) (Fig. 8B). In *B. cinerea*, mean hyphal length in the mock control (84.4 ± 10.6 µm) was significantly reduced to 50.6 ± 9.2 µm with 25% filtrate and to 28.5 ± 6.7 µm with 50% filtrate, corresponding to 40.0% and 66.2% inhibition, respectively. *A. niger* exhibited a similar dose-dependent trend, with hyphal lengths of 86.5 ± 12.3 µm (mock), 54.4 ± 8.8 µm (25%; 37.1% reduction), and 31.3 ± 5.9 µm (50%; 63.8% reduction). All reductions were statistically significant, confirming robust early-stage suppression of hyphal development. These observations provide mechanistic continuity with the colony-level inhibition shown in Fig. 6 and the reductions in spore viability and mycelial biomass shown in Fig. 7, indicating that Tch36 affects fungal development at multiple hierarchical stages, from spore germination and early hyphal emergence to subsequent colony establishment. Such stage-spanning inhibitory effects are consistent with previously reported modes of action for chitinase-mediated antifungal systems. The developmental inhibition observed here is compatible with the requirement for localized chitin remodeling during germination, where controlled cell-wall loosening and expansion are necessary for germ-tube emergence; interference with these processes is therefore expected to restrict fungal progression at an early developmental stage, prior to effective substrate colonization or host penetration.

Importantly, these inhibitory effects were achieved using crude, unpurified culture filtrates, yet the magnitude of suppression was comparable to that reported for purified fungal or bacterial chitinases (Hassan et al. 2009; Li et al. 2018; Deng et al. 2019; Abdelraouf et al. 2024). This observation underscores the functional competence of the *S. cerevisiae* secretion platform and highlights its practical relevance for scalable biocontrol formulations. From an applied perspective, early-stage inhibition is particularly advantageous, as limiting spore germination and initial hyphal growth substantially reduces the likelihood of disease establishment and subsequent spread.

Taken together with the colony-level and biomass-level inhibition described in preceding sections, these early-stage effects support a consistent inhibitory framework in which Tch36 interferes with fungal development across multiple developmental stages. Such multilayered interference supports the potential application of Tch36-producing *S. cerevisiae* strains, or their culture filtrates, as promising antifungal resources in agricultural and bioprocessing applications.

### Evidence of cell wall degradation via protoplast formation

**Fig. 9:**
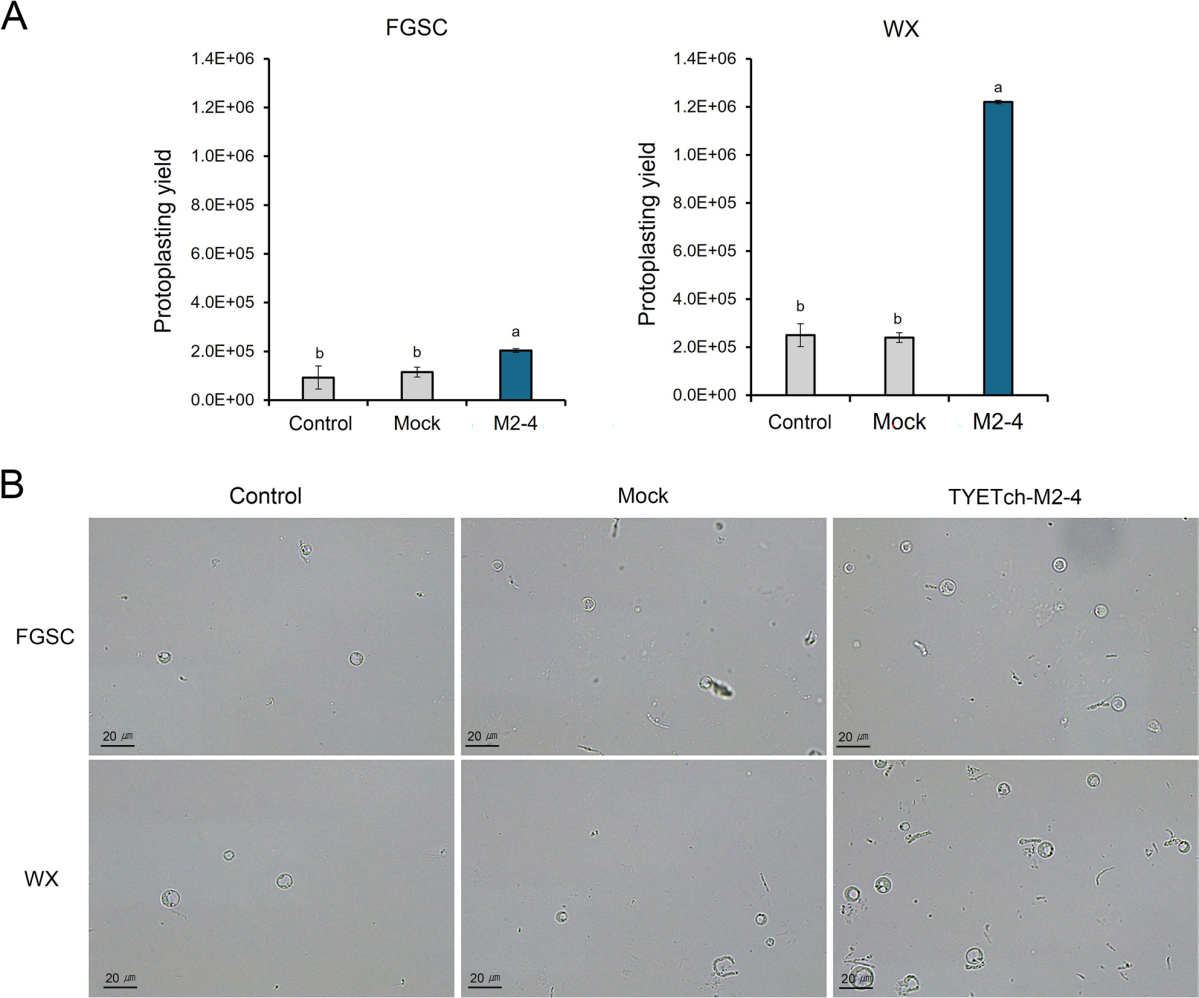
Effects of recombinant chitinase on protoplast formation in *Aspergillus nidulans*. Protoplast formation was assessed in two *A. nidulans* strains: **A** Fungal Genetics Stock Center (FGSA) A4 wild-type strain and WX mutant featuring a defective cell wall. Three treatment conditions were tested: (a) buffer supplemented with uninoculated culture medium (control), (b) buffer supplemented with culture filtrate from a mock *S. cerevisiae* strain carrying the empty vector (Mock), and (c) buffer supplemented with culture filtrate from recombinant *S. cerevisiae* strain TYETch-M (M2-4) expressing the *tch36* gene. **B** Protoplasts were visualized under a light microscope, and formation efficiency was quantified. All treatments were conducted under identical conditions. Error bars represent standard deviations from three independent biological replicates conducted on separate days (*n* = 3), each including three parallel protoplasting reactions per condition. Statistical significance was determined by one-way ANOVA followed by Duncan’s multiple range test (*p* < 0.05). Enhanced protoplast formation in the presence of recombinant filtrate suggests cell wall alteration mediated by secreted Tch36

To obtain direct mechanistic evidence that secreted Tch36 targets fungal cell walls, we performed protoplast formation assays using two *A. nidulans* strains: FGSC A4 (wild type) and WX, a mutant with a defective cell wall carrying the *npgA1* allele, which has been previously characterized as exhibiting defects in phosphopantetheinyl transferase activity, secondary metabolism, and cell wall–associated developmental processes (Márquez-Fernández et al. 2007; Kim et al. 2015). Three buffer conditions were compared: a broth-based control, a mock control containing supernatant from the empty-vector transformant (Mock) strain, and an experimental buffer supplemented with culture supernatant from the recombinant M2-4 strain expressing *tch36* (Fig. 9A, B). Importantly, all three buffers were supplemented with an identical basal protoplasting enzyme mixture, ensuring that any differences in protoplast yield reflect the additional contribution of secreted Tch36 rather than variation in background enzymatic activity.

Quantitative protoplast yields are shown in Fig. 9 A. In FGSC A4, M2-4 treatment generated an average of 2.03 × 10^5^ protoplasts, an approximately 2.2-fold and 1.8-fold increase relative to the broth (9.25 × 10^4^) and mock (1.15 × 10^5^) controls, respectively. These results indicate that extracellular Tch36 significantly enhanced protoplast formation even in wild-type cells with intact walls. As expected, the WX mutant, characterized by reduced β-1,3-glucan and chitin content, exhibited markedly higher protoplast release across all conditions. Under M2-4 treatment, protoplast yield reached 1.22 × 10^6^, corresponding to 4.9-fold and 5.1-fold increases relative to broth (2.50 × 10^5^) and mock (2.40 × 10^5^), respectively. Notably, protoplast production in WX under Tch36 exposure was nearly six-fold higher than in FGSC A4, underscoring the dependence of Tch36 activity on cell wall accessibility. Qualitative microscopy observations further supported these trends. Samples treated with M2-4 displayed markedly higher numbers of spherical, wall-less protoplasts, whereas broth and mock treatments showed fewer or partially lysed cells. These visual data corroborate the quantitative results, affirming that Tch36 actively degraded structural components of the cell wall.

These findings are consistent with previous reports that chitinases facilitate protoplast formation by enzymatically weakening chitin-rich wall layers (Hassan et al. 2009, 2014; Wu and Chou 2019). Importantly, under identical protoplasting conditions employing VinoTaste Pro, supplementation with recombinant M2-4 culture filtrate resulted in a statistically significant increase in protoplast yield relative to the mock control. These data demonstrate that extracellular Tch36 enhances fungal cell wall susceptibility within the assay system. Because the commercial enzyme preparation provides baseline wall-degrading activity, the increased protoplast formation reflects a functional contribution of the recombinant filtrate beyond the activity supplied by the commercial enzyme alone. However, the relative contributions of additive enzyme dosage effects versus potential synergistic interactions with components of the commercial enzyme mixture cannot be fully distinguished within the current assay design (Wang et al. 2020a, b; Subramanian et al. 2020).

**Fig. 10:**
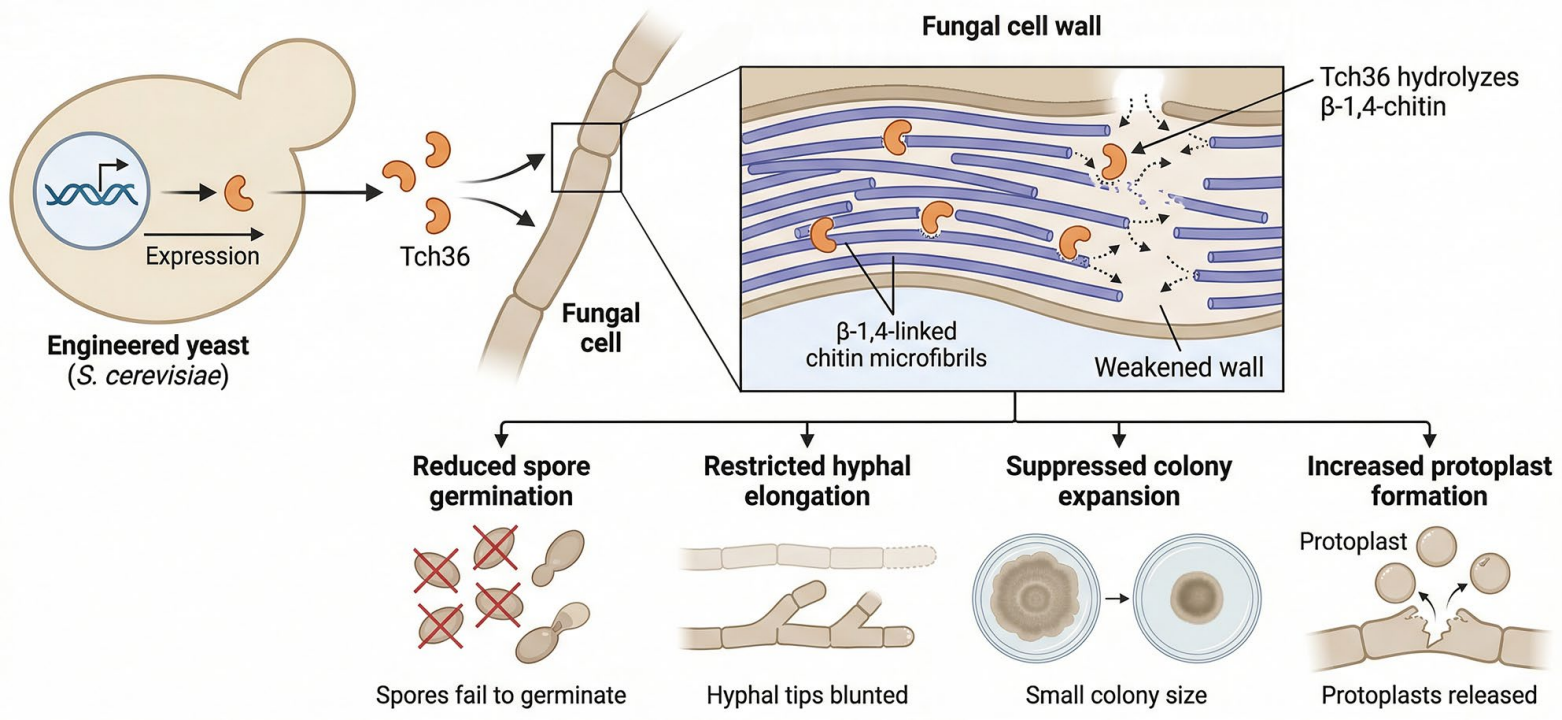
Proposed multistage cell wall–targeting model of yeast-secreted Tch36. Engineered *S. cerevisiae* secretes extracellular Tch36, which hydrolyzes β-1,4-linked chitin microfibrils within the fungal cell wall. This activity is proposed to weaken the structural wall matrix and is associated with reduced germ tube emergence, limited hyphal elongation, suppressed colony expansion, and enhanced protoplast formation. The schematic integrates the experimental observations presented in Fig. 6–9. This schematic summarizes the experimental observations and presents a proposed multistage model of cell wall targeting by yeast-secreted Tch36

Taken together, these findings indicate that yeast-secreted Tch36 compromises fungal cell wall integrity, consistent with established models of chitinase-mediated antagonism (Harman 2006) and with the observed reductions in colony expansion, spore viability, early hyphal elongation, and enhanced protoplast formation described above. The combined data can be integrated into a proposed cell wall–targeting framework (Fig. 10), in which extracellular Tch36 hydrolyzes β-1,4-linked chitin microfibrils, contributing to localized weakening of the wall matrix. Such destabilization is expected to interfere with cell wall remodeling during germ tube emergence and apical extension, thereby restricting early developmental progression and colony establishment. The increased protoplast yield under identical basal enzyme conditions further supports enhanced wall susceptibility in the presence of Tch36.

## Conclusion

This study establishes a robust heterologous secretion platform in the GRAS yeast Saccharomyces cerevisiae for the extracellular production of the Trichoderma atroviride chitinase Tch36 under laboratory-scale conditions. By combining the rice α-amylase signal peptide with either full-length or mature *tch36* coding sequences under the constitutive GPD promoter, we successfully generated recombinant yeast strains capable of secreting catalytically active chitinase directly into the culture medium. Notably, the mature construct consistently outperformed the full-length version in terms of transcriptional output, secretion efficiency, and enzymatic activity, underscoring the benefit of removing the native fungal signal peptide for optimal heterologous expression in yeast.

Crude, non-purified culture filtrates from optimized transformants exhibited broad antifungal activity against diverse plant pathogenic fungi as well as opportunistic *Aspergillus* species. The inhibitory effects were evident across multiple stages of fungal development, including spore germination, early hyphal growth, and colony establishment. Protoplast formation assays further supported a direct mode of action targeting the fungal cell wall, providing mechanistic evidence for the biological efficacy of yeast-secreted Tch36.

Importantly, all observed antifungal and protoplasting activities were achieved without enzyme purification, highlighting a production process that is purification-free and operationally straightforward under laboratory-scale conditions. Given the established safety profile and bioprocessing versatility of *S. cerevisiae*, the platform described here provides a functional foundation for the development of chitinase-based biocontrol agents. By coupling a plant-derived secretion signal with a fungal chitinase, this work addresses a key limitation in chitinase biomanufacturing—namely inefficient heterologous secretion and reliance on downstream purification steps—thereby advancing yeast-based chitinase production beyond proof-of-concept demonstration. Future studies focusing on quantitative productivity benchmarking, process optimization, formulation strategies, enzyme stabilization, and in planta validation will further clarify the translational potential of this system in sustainable agricultural biotechnology and microbial bioprocessing.

## Supplementary Information

Below is the link to the electronic supplementary material.

Supplementary Material 1.

## Data Availability

The nucleotide sequence data generated in this study have been deposited in GenBank under accession number **OM240654**. All other data supporting the findings of this study are available from the corresponding author upon reasonable request.
