# Supplementary material for: Purification-free antifungal biocontrol platform using engineered Saccharomyces cerevisiae secreting Trichoderma atroviride chitinase
Source: Bioresour Bioprocess. 2026 Apr 29;13(1):61. doi: 10.1186/s40643-026-01054-z (PMC13129169; doi:10.1186/s40643-026-01054-z)
Supplement: Supplementary file 1 — Supplementary Material 1. [file 40643_2026_1054_MOESM1_ESM.docx]

**Supplementary Information**

**Purification-free antifungal biocontrol platform using engineered *Saccharomyces cerevisiae* secreting *Trichoderma atroviride* chitinase**

Ha-Yeon Song^1^, Dae-Hyuk Kim^2^, Jung-Mi Kim ^3,*^, and Ji Young Kang^4,*^

**Supplementary Table 1. Primer sequences used for cDNA amplification, qRT-PCR, and fusion PCR**

| Primer | Sequence (5'→3') | Purpose | Length (bp) |
| --- | --- | --- | --- |
| Ta-chit36F | CGG GAT CCA TGA CAC GCC TTC TTG ACG CC | cDNA amplification | 29 |
| Ta-chit36R | TCC CCC GGG CTA ACC AAT GCG AGT AAG CAA GTT GTC |  | 36 |
| asp-F | CGG GAT CCA TGC AGG TGC TGA ACA C | Fused PCR | 25 |
| asp-tchF-R | GGC GTC AAG AAG GCG TGT CAT CCC GGC TGT CAA GTT A |  | 37 |
| asp-tchM-R | CTT GAG TGC ACA TGT TGC ATT TTG CCC GGC TGT CAA GTT A |  | 40 |
| Ftch36F-F | TAA CTT GAC AGC CGG GAT GAC ACG CCT TCT TGA CGC C |  | 37 |
| Ftch36F-R | CGC GTC GAC CTA ACC AAT GCG AGT AAG CAA GTT GTC |  | 36 |
| Ftch36M-F | TAA CTT GAC AGC CGG GCA AAA TGC AAC ATG TGC ACT CAA G |  | 40 |
| GPD-RTF | TCT CCT CTG ACT TCT TGG | qRT-PCR | 18 |
| GPD-RTR | CGT ATT CGT TGT CGT ACC |  | 18 |
| Tch36-RTF | ATC ATA CCT GCC AAT CAT TC |  | 20 |
| Tch36-RTR | CTG AGC AAC CGT AGT AGT |  | 18 |

**Supplementary Table 2. Molecular characteristics and sequence comparison of the *tch36* gene from *T. atroviride*.**

| Feature | Description |
| --- | --- |
| Gene name | *tch36* (this study) |
| Source organism | *Trichoderma atroviride* KACC 40774 |
| Gene length | 1035 bp (cDNA) |
| Predicted protein length | 344 amino acids^†^ |
| GenBank accession | OM240654^*^ |
| Nucleotide identity | 98.0% compared with *chit36* from *T. atroviride* (GenBank: KT992143) |
| Amino acid identity | 99.1% compared with *chit36* from *T. atroviride* (GenBank: KT992143) |

* GenBank accession number is pending submission.

† Protein length includes signal peptide.


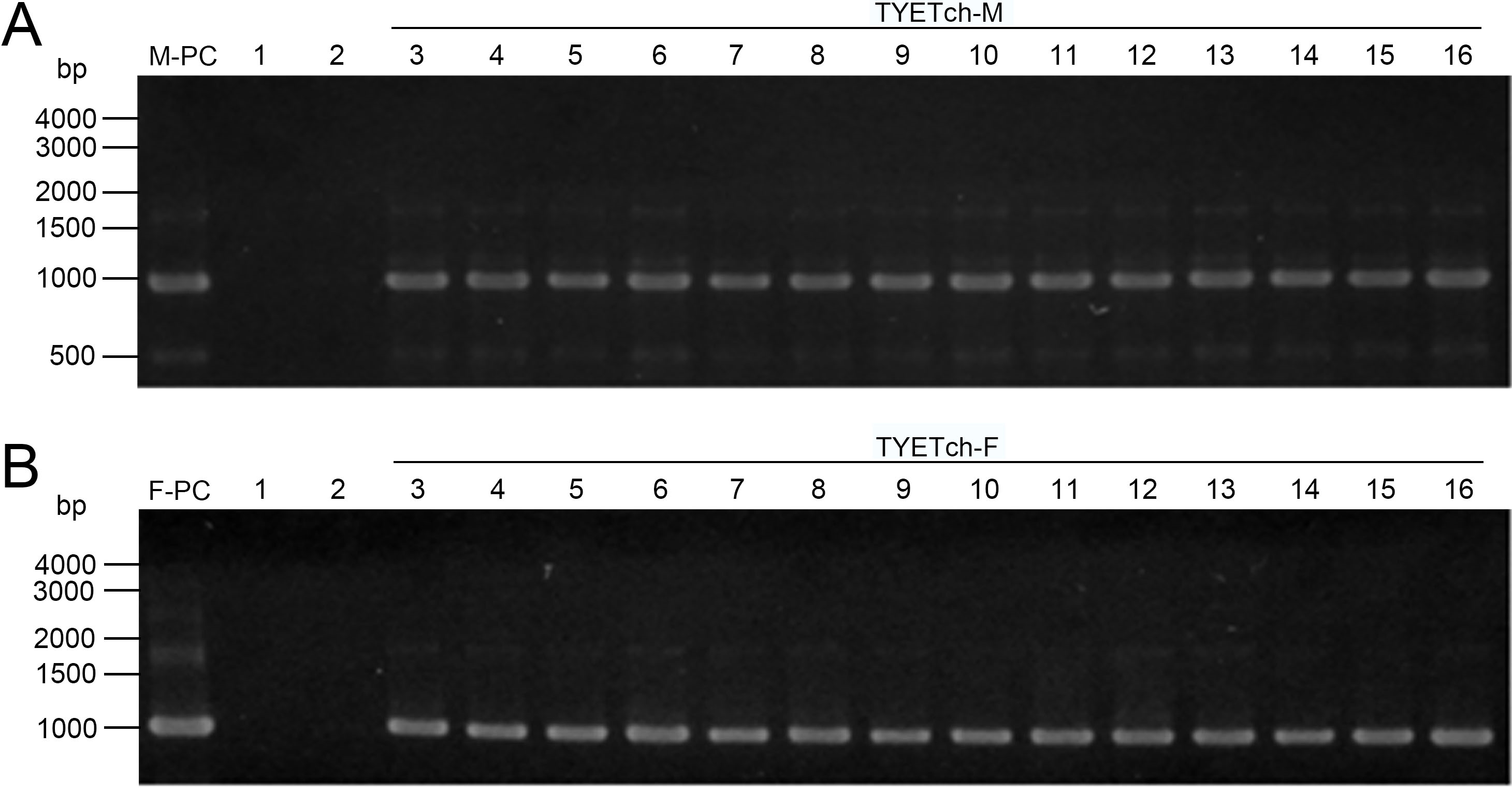


**Supplementary Figure 1. Colony PCR amplification of the *tch36* gene from *T. atroviride*.** Gel electrophoresis analysis of colony PCR products from the Y2805 yeast strain transformed with pYETch36M (M-PC, panel A) or pYETch36F (F-PC, panel B). PCR was performed using 1% agarose gel. Lane M-PC or F-PC contains PCR products amplified from the corresponding plasmid DNA used for yeast transformation, serving as positive controls. Lanes 1 and 2 show results from the recipient strain and the mock transformant (vector only), respectively, serving as negative controls. Lanes 3–16 show PCR products from fourteen independent TYETch-M (A) or TYETch-F (B) transformants. Molecular size markers are indicated on the left. These PCR results confirm successful integration of the tch36 gene in the selected yeast transformants.


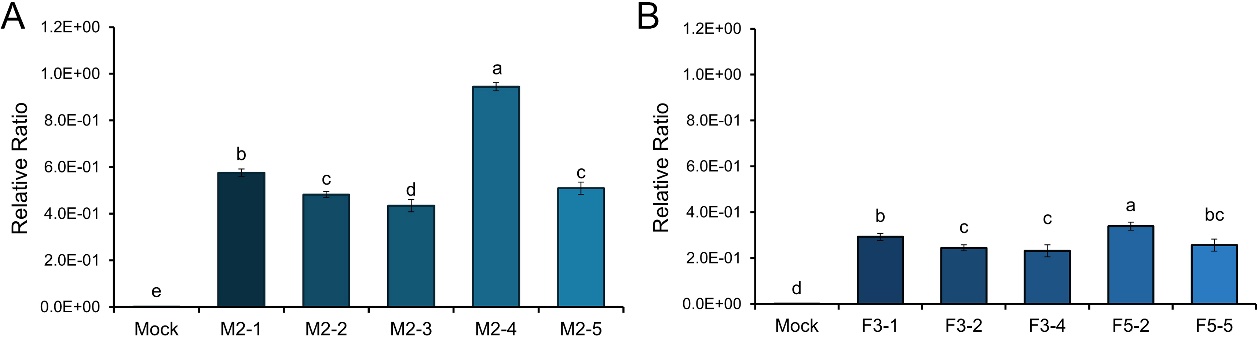


**Supplementary Figure 2. Relative expression of the T. atroviride tch36 gene in recombinant S. cerevisiae.** The expression levels of the tch36 gene from T. atroviride were quantified by quantitative real-time PCR (qRT-PCR) using total RNA extracted from recombinant yeast strains **TYETch-M (A)** and **TYETch-F (B).** Transcript levels were normalized relative to the expression of the endogenous S. cerevisiae glyceraldehyde-3-phosphate dehydrogenase **(GPD)** gene. **The y-axis represents expression levels relative to the GPD gene. Light gray bars indicate the empty vector control (Mock).** Blue tone bars represent individual recombinant strains: M2-1, M2-2, M2-3, M2-4, and M2-5 for TYETch-M (panel A), and F3-2, F3-3, F3-4, F5-2, and F5-5 for TYETch-F (panel B). Error bars represent mean ± standard deviation (SD) from three independent biological replicates (n = 3), in which total RNA was independently extracted and each qRT-PCR reaction was performed in technical triplicate. Statistical significance was determined using one-way ANOVA followed by Duncan’s multiple range test (*p* < 0.05). These PCR results verify the presence of the inserted tch36 gene in the analyzed yeast transformants.


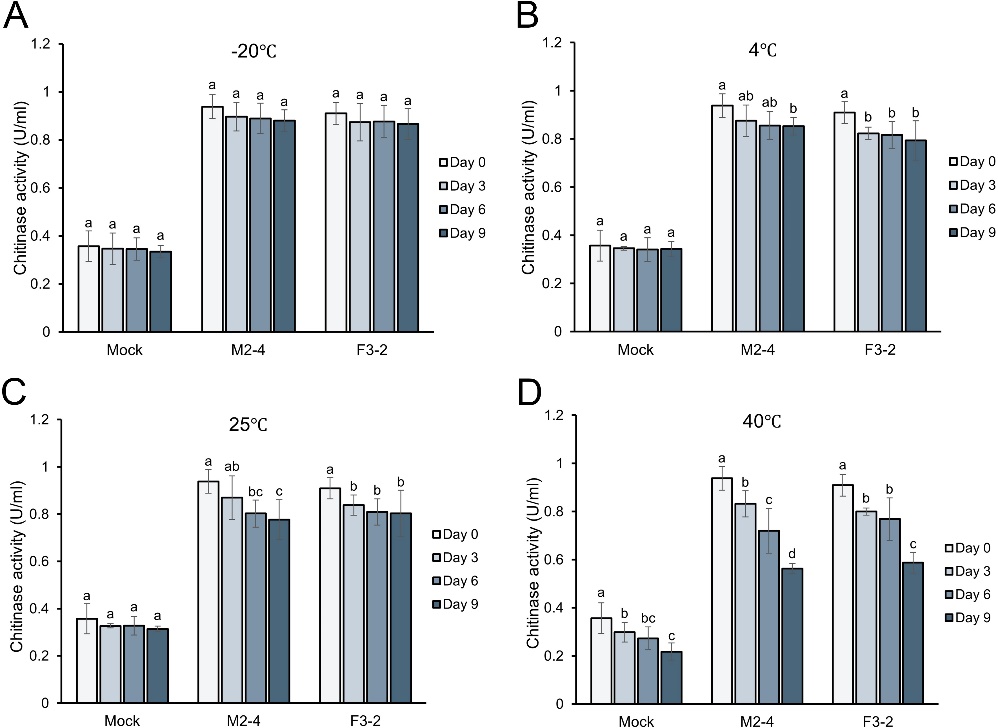


.

**Supplementary Figure 3. Temperature-dependent storage stability of chitinase activity in recombinant *S. cerevisiae*.** Chitinase activities of Mock, M2-4, and F3-2 strains were measured after storage at −20 °C (A), 4 °C (B), 25 °C (C), and 40 °C (D) for 0, 3, 6, and 9 days. Enzyme activity is expressed as U/mL. Light gray bars represent the Mock control, and blue-toned bars represent the recombinant strains (M2-4 and F3-2). Data are presented as mean ± standard deviation (SD) from three independent biological replicates (n = 3). Statistical significance was determined by one-way ANOVA (*p* < 0.05), and different lowercase letters indicate significant differences among storage time points within each strain at the same temperature. Overall, recombinant strains retained substantial chitinase activity under low-temperature storage, whereas progressive activity reduction was observed at elevated temperatures.


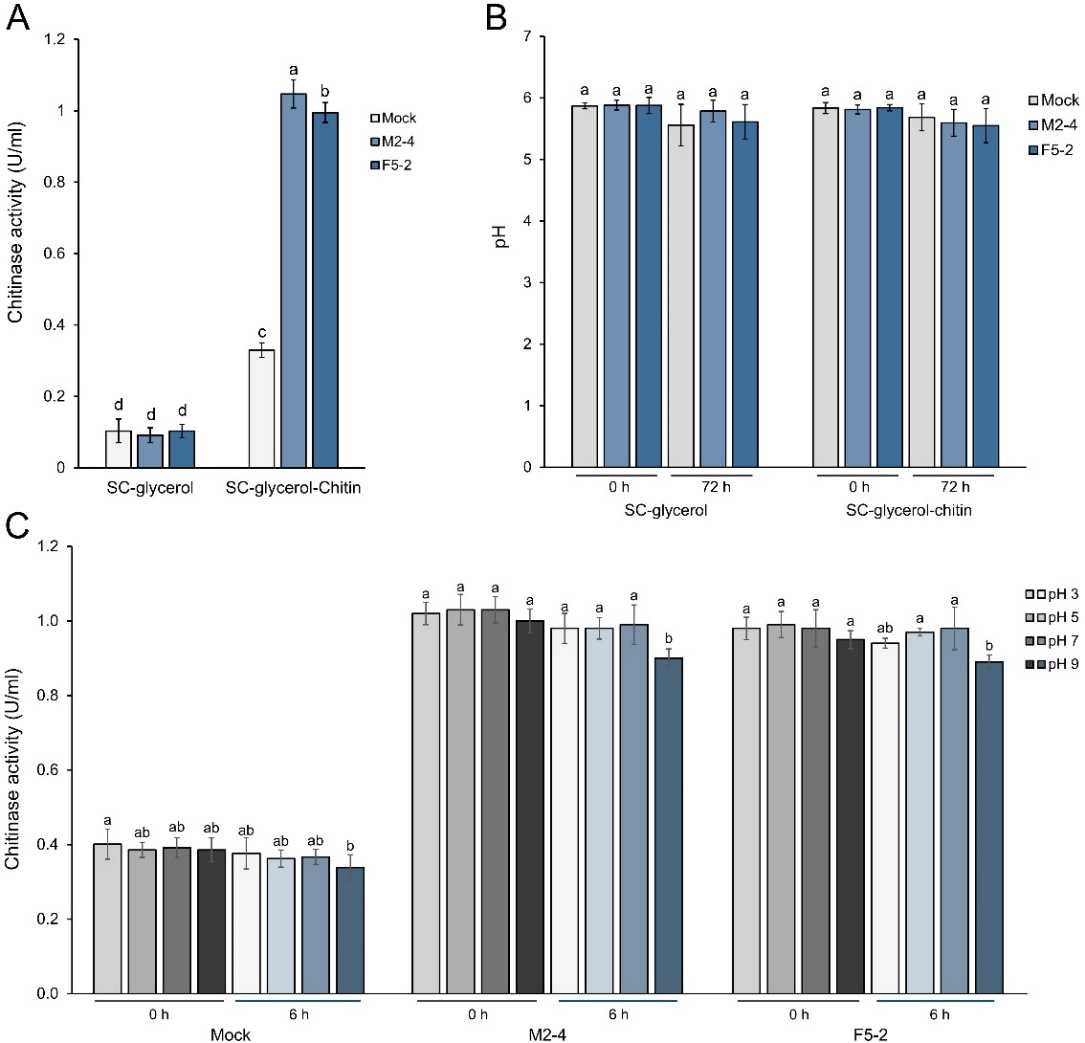


**Supplementary Figure 4. Colloidal chitin-dependent induction and pH stability profile of recombinant chitinase.** (A) Chitinase activity (U/mL) of Mock, M2-4, and F5-2 strains cultured in SC-glycerol medium with or without colloidal chitin supplementation. (B) Culture supernatant pH measured at 0 h and after 72 h incubation in SC-glycerol and SC-glycerol–chitin media. (C) Residual chitinase activity (U/mL) measured at pH 3, 5, 7, and 9 immediately after pH adjustment (0 h) and after 6 h incubation to assess short-term pH stability. Data are presented as mean ± standard deviation (SD) from three independent biological replicates (n = 3). Statistical significance was determined by one-way ANOVA (*p* < 0.05), and different lowercase letters indicate significant differences within each strain under the same experimental condition. Overall, colloidal chitin supplementation enhanced chitinase production in recombinant strains, while enzymatic activity remained relatively stable across neutral to mildly acidic conditions.

**Supplementary Table 3. Quantitative data underlying the antifungal activity assays shown in Figure 6.**

| Strain | Relative Activity (%) $\pm$SD^*^ | | | |
| --- | --- | --- | --- | --- |
|  | Mock | TYETch-M2-4 | TYETch-F3-2 |  |
| *A. alternata* | 100^a^ | 79.82$\pm$0.78^b^ | 82.96$\pm$1.55^c^ |  |
| *B. cinerea* | 100^a^ | 67.44$\pm$2.13^b^ | 69.30$\pm$1.61^b^ |  |
| *B. dothidea* | 100^a^ | 77.73$\pm$3.21^b^ | 72.47$\pm$3.21^c^ |  |
| *C. acutatum* | 100^a^ | 83.09$\pm$2.55^b^ | 88.24$\pm5.84$^b^ |  |
| *F. fujikuroi* | 100^a^ | 76.03$\pm$1.89^b^ | 76.45$\pm$1.43^b^ |  |
| *F. graminearum* | 100^a^ | 51.59$\pm$4.96^b^ | 57.14$\pm$1.19^b^ |  |
| *R. solani* | 100^a^ | 75.53$\pm$2.13^b^ | 74.82$\pm$2.21^b^ |  |
| *T. atroviride* | 100^a^ | 84.34$\pm$1.20^b^ | 86.35$\pm$1.84^b^ |  |
| *T. viride* | 100^a^ | 79.12$\pm$1.39^b^ | - 1. $\pm$0.70^c^ |  |

^*^ Data are mean ± SD from three independent biological replicates (n = 3), each performed with five technical replicates per condition. Statistical analysis was conducted using one-way ANOVA followed by Duncan’s multiple range test (*p* < 0.05). Means sharing the same letter within a column are not significantly different.
